# Impact of Cell Composition and Geometry on Human Induced Pluripotent Stem Cells-Derived Engineered Cardiac Tissue

**DOI:** 10.1038/srep45641

**Published:** 2017-04-03

**Authors:** Takeichiro Nakane, Hidetoshi Masumoto, Joseph P. Tinney, Fangping Yuan, William J. Kowalski, Fei Ye, Amanda J. LeBlanc, Ryuzo Sakata, Jun K. Yamashita, Bradley B. Keller

**Affiliations:** 1 Kosair Charities Pediatric Heart Research Program, Cardiovascular Innovation Institute, University of Louisville, Louisville, Kentucky, The United States of America; 2 Department of Cell Growth and Differentiation, Center for iPS Cell Research and Application (CiRA), Kyoto University, Kyoto, Japan; 3 Department of Cardiovascular Surgery, Kyoto University Graduate School of Medicine, Kyoto, Japan; 4 Department of Pediatrics, University of Louisville School of Medicine, Louisville, Kentucky, The United States of America; 5 Department of Physiology, University of Louisville, Louisville, Kentucky, The United States of America

## Abstract

The current study describes a scalable, porous large-format engineered cardiac tissue (LF-ECT) composed of human induced pluripotent stem cells (hiPSCs) derived multiple lineage cardiac cells with varied 3D geometries and cell densities developed towards the goal of scale-up for large animal pre-clinical studies. We explored multiple 15 × 15 mm ECT geometries using molds with rectangular internal staggered posts (mesh, ME), without posts (plain sheet, PS), or long parallel posts (multiple linear bundles, ML) and a gel matrix containing hiPSC-derived cardiomyocytes, endothelial, and vascular mural cells matured *in vitro* for 14 days. ME-ECTs displayed the lowest dead cell ratio (p < 0.001) and matured into 0.5 mm diameter myofiber bundles with greater 3D cell alignment and higher active stress than PS-ECTs. Increased initial ECT cell number beyond 6 M per construct resulted in reduced cell survival and lower active stress. The 6M-ME-ECTs implanted onto 1 week post-infarct immune tolerant rat hearts engrafted, displayed evidence for host vascular coupling, and recovered myocardial structure and function with reduced scar area. We generated a larger (30 × 30 mm) ME-ECT to confirm scalability. Thus, large-format ECTs generated from hiPSC-derived cardiac cells may be feasible for large animal preclinical cardiac regeneration paradigms.

Heart diseases are the leading cause of death worldwide. Even with a broad range of evidence-based therapies, the five-year survival rate of heart failure remains as low as approximately 50%^1^. Numerous preclinical studies and clinical trials have suggested that application of stem and/or progenitor cell populations to an injured heart may hold a potential to ameliorate left ventricular dysfunction caused by ischemic and dilated cardiomyopathy accompanying heart failure^2^,^3^,^4^,^5^,^6^,^7^,^8^. Among various cell types, human induced pluripotent stem cells (hiPSCs) are considered highly promising cell sources for cardiac regenerative cell therapy, because the fundamental etiology of heart failure is the result of massive loss or dysfunction of myocardial cells^9^, and various cardiovascular (CV) cell lineages can be scalably produced from iPSCs^10^,^11^,^12^.

Tissue engineering technologies have emerged as robust modalities to realise cardiac regeneration due to the unique capacity to deliver numerous cardiac cells within an organised architecture onto the heart^13^,^14^,^15^,^16^,^17^. Previously, we reported the generation of three-dimensional (3D) linear engineered cardiac tissues (ECTs) from chick embryonic or rat fetal cardiomyocytes (CMs) and biomaterials as a robust *in vitro* model to elucidate the development of embryonic myocardium and a platform to realise cardiac regeneration via implantation therapy for injured myocardium^18^,^19^. In order to advance this technology towards clinical application, we developed and validated a method to generate linear ECTs from human iPSCs-derived CV lineages (hiPSC-ECTs)^8^. There we found that coexistence of multiple vascular lineages with CMs within the 3D ECT composition promoted structural and electrophysiological tissue maturation. Furthermore, we demonstrated the therapeutic potential of hiPSC-ECTs in an immune tolerant rat myocardial infarction (MI) model showing the improvement of cardiac function with regenerated myocardium and enhanced angiogenesis.

In the current study we describe the development of a larger implantable tissue which provides the framework for scale-up to pre-clinical studies using large animal models with human-sized hearts and eventual clinical studies. Several ECT scale-up strategies have been described including pre-vascularization^20^,^21^, stacking cell sheets^22^, scalable scaffolds^23^, and bioprinting^24^. Guided by initial works from the Bursac lab using Polydimethylsiloxane (PDMS) molds to form porous engineered tissues from neonatal rat skeletal myoblasts and CMs^25^, human ESC-derived CMs^26^, and mouse iPSCs^27^, we fabricated a range of mold geometries from 0.5mm thick PDMS sheets. We have expanded our hiPSC-ECT technology to develop a novel large-format hiPSC-ECT (LF-ECT) through optimisation of geometry and cellular composition in order to promote *in vitro* pre-implant cell survival and satisfactory engraftment after *in vivo* implantation onto animal hearts.

## Results

**Figure 1:**
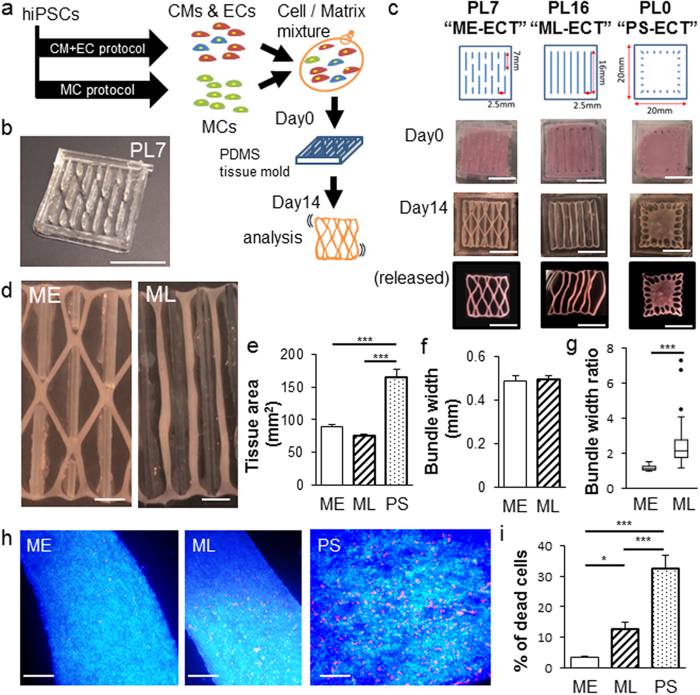
Impact of initial geometry on LF-ECT maturation and cell survival. (**a**) Schematic diagram for generation of LF-ECTs containing cardiomyocytes (CM), endothelial cells (EC), and mural cells (MC). Cells and matrix were poured into custom PDMS molds on day 0 and then matured *in vitro*. (**b**) Representative image of a LF-ECT mold constructed from 0.5 mm diameter PDMS sheets. Scale bar: 10 mm. (**c**) Representative initial LF-ECT geometries. PL7 mold (ME-ECT) included post lengths of 7 mm and post spacing of 2.5 mm. PL16 mold (ML-ECT) included post lengths of 16 mm and post spacing of 2.5 mm. PL0 mold (PS-ECT) included individual 1 mm diameter loading posts located along the LF-ECT periphery. Representative images of LF-ECTs on day 0, day 14, and after release from the mold are shown. Scale bar: 10 mm. (**d**) Representative image of a day 14 ME-ECT that shows the mature bundles and junctions and a day 14 PL-ECT that shows variation in bundle width. Scale bar: 2.5 mm. (**e**) Cross-sectional tissue area was increased in PS-ECT (n = 8) versus ME- (n = 10) and ML-ECTs (n = 6) (***P < 0.001 for each). (**f**) Bundle widths were similar between ME- (n = 10) and ML-ECTs (n = 6). (**g**) The ratio of maximum to minimum width of each bundle was calculated, and lower variance was noted in ME- versus ML-ECTs (n = 36; ***P < 0.001). (**h**) Representative images for ME-, ML-, and PS-ECTs stained with EthD-III (red) for dead cells. Scale bar: 250 μm. (**i**) ME-ECTs had a smaller percent of dead cells at day 14 versus ML- and PS-ECTs (n = 20; *P < 0.05, ***P < 0.001).

We induced multiple CV cell lineages from hiPSCs to generate LF-ECTs using the lineage distribution shown to generate an optimal linear hiPSC-derived ECTs^8^. We employed two distinct CV cell differentiation protocols to generate either predominantly cardiac troponin-T (cTnT)^+^ CMs and vascular endothelial (VE)-cadherin (CD144)^+^ endothelial cells (ECs) or to generate predominantly platelet-derived growth factor receptor beta (PDGFRβ; CD140b)^+^ vascular mural cells (MCs) (Fig. 1a and Supplementary Figure 1a). We then mixed induced CV cells from these two protocols to adjust final EC and MC concentrations to represent 10 to 20% of total cells to facilitate the *in vitro* expansion of vascular cells within ECTs and subsequent *in vivo* vascular coupling between ECTs and recipient myocardium (Fig. 1a). The calculated composition of CMs, ECs, and MCs for ECT preparation was 44.2 ± 0.6%, 15.9 ± 0.5%, and 13.0 ± 0.4%, respectively (n = 107 constructs)(Supplementary Figure 1b). The cellular component of TRA-1-60-positive undifferentiated hiPSCs within cell mixtures used for each ECT was 5.4 ± 0.1% (n = 107). We also quantified the percentage of the additional mesenchymal surface markers CD44 and CD90 (Thy1) (10.2 ± 1.3% and 11.2 ± 0.2%, respectively, n = 3).

We generated LF-ECTs containing CM + EC + MC cells harvested on d15, collagen I, and Matrigel as previously described^8^. In order to investigate the relationship between LF-ECT geometry and LF-ECT structural and functional maturation we fabricated tissue molds with distinct patterns characterised by various post lengths and spacing (Fig. 1b and Supplementary Figure 2). We prepared three general categories of tissue molds: 1) molds with 7 mm post lengths, (PL) arrayed at a staggered position to facilitate formation of a mesh structure with bundles and junctions (PL7, ME-ECT); 2) molds with multiple 16 mm long posts to support formation of parallel linear bundles without junctions (PL16, ML-ECT); and 3) molds with peripheral anchors but no internal posts to generate a plain sheet without central pores (PL0, PS-ECT) (Fig. 1c). During preliminary experiments, we noted that ECTs adhered to the PDMS posts during gel compaction and so we modified our protocol to coat each mold with Pluronic F127 prior to use in order to prevent cell adhesion^25^. Similar to linear ECTs, each construct exhibited rapid gel compaction and acquired characteristic geometries dependent on the mold design (Fig. 1c,d). Those structures were maintained even after released from tissue molds (Fig. 1c). All constructs started intrinsic spontaneous beating *in vitro* within 3 days and then continued beating throughout the duration of culture. We measured ECT cross-sectional surface area after ECT formation on day 0 and at intervals for two weeks of culture to calculate gel compaction during tissue maturation. Following gel compaction, d14 ME-ECTs and ML-ECTs tissue areas were comparable and significantly less than d14 PS-ECTs [89.5 ± 3.1 mm^2^ (n = 10) and 75.6 ± 1.6 mm^2^ (n = 6), versus 165.9 ± 11.0 mm^2^ (n = 8), respectively, (P < 0.001, Fig. 1e)]. The mean bundle widths in ME-ECTs and ML-ECTs were comparable [0.49 ± 0.08 mm (n = 10) and 0.49 ± 0.04 mm (n = 6), Fig. 1f], however, ML-ECTs showed greater variation in bundle width likely due to substantially longer bundle lengths (P < 0.001, Mann-Whitney U test, Fig. 1g). We determined the impact of initial geometry on d14 cell viability using Live/Dead assay (Fig. 1h) and found that cell viability was greatest in ME-ECTs and lowest in PS-ECTs (Fig. 1i), indicating that both the presence of pores and ME geometry facilitated *in vitro* LF-ECT cell survival. Of note, the greater cell death noted in ML-ECTs than ME-ECTs could be due to greater variations in bundle widths in ML-ECTs, greater variations in bundles stresses due to variations in bundle widths, and/or the tendency for part of the ML-ECTs to rest on the bottom of the culture tray, possibly reducing buffer and nutrient delivery to these segments.

**Figure 2:**
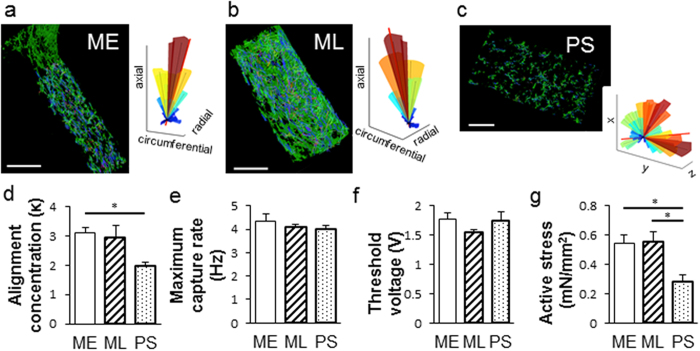
Impact of initial geometry on LF-ECT cardiomyocyte alignment and contractile function. Representative alignment analysis using whole-mount cTnT-stained images. (**a**) ME-ECT, (**b**) ML-ECT, (**c**) PS-ECT. Summary 3D alignment data for the adjacent image is shown with construct long axis identified (red line). (**d**) Note increased dispersion of CM orientation in PS-ECT 3D plot, quantified by reduced alignment concentration (κ), (n = 4; *P < 0.05 for PS versus ME). (**e**) Maximum capture rate, and (**f**) excitation thresholds, were similar between the three geometries [n = 11 (ME), 11 (ML), and 8 (PS)]. (**g**) Active stress under 2 Hz/5 V electrical pacing was lower in PS-ECT (n = 8) versus ME- and ML-ECTs (n = 11; *P < 0.05 for PS versus ME or ML).

We next determined the relationship between LF-ECT geometry and 3D CM alignment using whole mount confocal imaging and a custom 3D cell orientation analysis program (see Supplementary Methods). We reconstructed sequential cardiac troponin-T (cTnT) immunostained 2D confocal microscopic image stacks and then calculated myofiber orientation within each geometric class of LF-ECT (Fig. 2a–c). ME-ECTs showed higher values for the concentration parameter (к) versus PS-ECTs (P < 0.05), representing greater myofiber alignment relative to the local bundle long axis (Fig. 2d). We then explored the impact of LF-ECT geometry on electromechanical properties using a custom intact muscle test system^8^,^18^,^19^. Maximum paced capture rates and excitation threshold voltages were similar among three groups (Fig. 2e,f), however, active stress at 2 Hz/5 V pacing in ME-ECTs and ML-ECTs was higher than in PS-ECTs (P < 0.05, Fig. 2g) consistent with greater cell viability and increased alignment versus PS-ECTs. Of note, maximum capture rates, excitation threshold voltages, and active stress for ME-ECTs and ML-ECTs were similar to comparable functional measures in hiPSC-derived (CM + EC + MC) linear ECTs in our previous study, consistent with comparable lineage compositions^8^.

**Figure 3:**
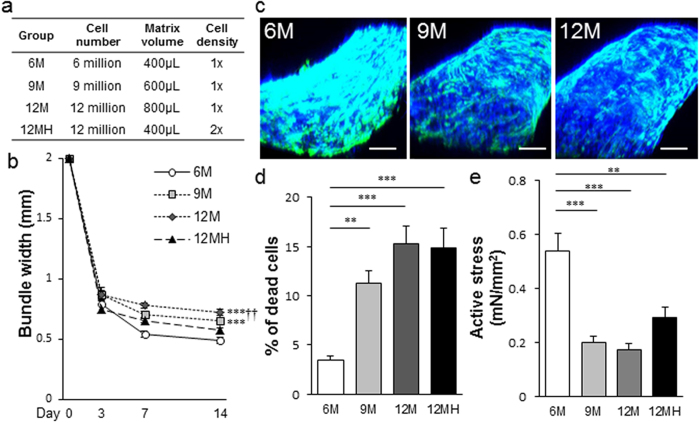
Impact of initial cell number and cell density on LF-ECT cell survival, maturation, and function for ME-ECTs. (**a**) We varied total cell number and volume from the standard 6 M in 400 μl to 9 M or 12 M with increasing initial volumes or to 12 M in the same initial volume (12 MH, high density). (**b**) Increased initial LF-ECT construct volumes resulted in larger bundle widths at d14 (***P < 0.001 for 9 M and 12 M versus 6 M; ^††^P < 0.01 for 12 M versus 12 MH) [n = 10 (6 M), 7 (9 M), 8 (12 M), and 7 (12 MH)]. (**c**) Representative 3D constructed tissue images for 6 M, 9 M, and 12 M ME-ECTs stained with DAPI and cTnT; cardiac Troponin T. Scale bar: 200 μm. (**d**) 6 M ME-ECTs had the smallest percent of dead cells at day 14 versus 9 M (**P < 0.01) and versus 12 M and 12 MH (***P < 0.001). N = 20. (**e**) Active stress under 2 Hz/5 V electrical pacing was highest in 6 M ME-ECT versus ECTs with greater initial cell numbers [n = 11 (6 M), 8 (9 M), 8 (12 M), and 8 (12 MH); ***P < 0.001 vs. 9 M and vs. 12 M, **P < 0.01 vs. 12 MH].

Once we determined the structural and functional advantage of the ME-ECT geometry, we then explored the impact of altered initial cell seeding number and seeding density on cell viability and *in vitro* LF-ECT structural and functional maturation. We generated ME-ECTs with increasing initial cell numbers [6 × 10^6^ (6 M), 9 × 10^6^ (9 M), or 12 × 10^6^ (12 M) cells] by using a standard cell density of 15 × 10^6^ cells/ml and increased initial cell/matrix volume from 400 μl to 600 μl or 800 μl and generated a fourth group with 2-fold higher cell density [12 × 10^6^ cells at 30 × 10^6^ cells/ml (12 MH)] in 400 μl (12 MH, Fig. 3a). The percentage of cellular components (CMs, ECs, MCs and other cells) in each group was comparable (Supplementary Table 1). In the standard density groups, 9 M and 12 M ME-ECTs exhibited wider bundles than 6 M ECTs and final bundle width positively correlated with initial seeding cell number and gel volume (P < 0.001, Fig. 3b). The 12 MH ME-ECTs also formed wider bundles at day 14 compared to the standard 6 M ME-ECTs despite the same initial gel volume, consistent with higher cell death and reduced gel compaction, though the difference was not statistically significant. Based on 3D histology, 9 M and 12 M ME-ECTs showed more sparsely distributed CMs, mainly located along the ME-ECT surfaces, compared to 6 M ECTs (Fig. 3c, Supplementary Video 1). We assessed the relationship between initial cell number and density on cell survival for each group (Fig. 3d). The percentage of dead cells on d14 in 9 M was more than three-fold greater than in 6 M (P < 0.01) with even higher dead cell percentages for the 12 M and 12 MH groups (P < 0.001), supporting the original 6 M maximal seeding number and density as optimal for the ME-ECT geometry. Consistent with the lower cell death of 6 M ME-ECTs, we noted the highest active stress under 2 Hz/5 V electrical pacing in 6 M ME-ECTs versus 9 M, 12 M (P < 0.001), and 12 MH (P < 0.01, Fig. 3e). Somewhat surprisingly, we found that increasing cell density from 15 × 10^6^ of 6 M ECTs to 30 × 10^6^ cells/ml of 12 MH ECTs produced lower active stress, suggesting that nutrient availability within the gel may impact cell survival and function.

**Figure 4:**
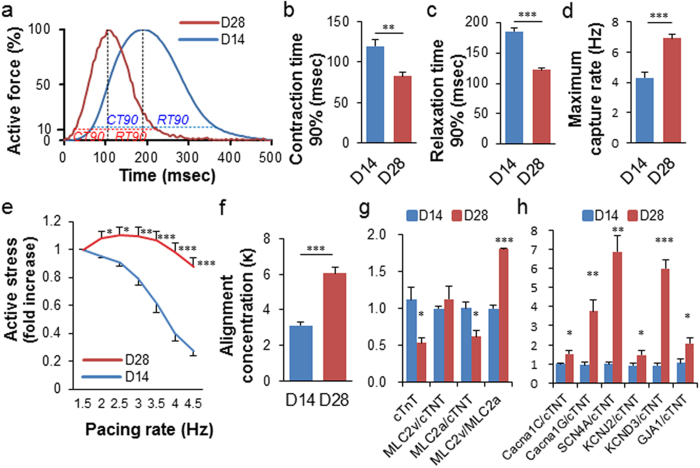
Impact of culture duration on ME-ECT maturation. (**a**) Averaged normalized active force-time curves for ME-ECT cultured for either day14 (blue) or day28 (red) (n = 6) under 2 Hz electrical stimulation. Dashed lines highlight 90% contraction time (CT) and relaxation time (RT). Prolonged *in vitro* culture for 28 days resulted in (**b**) reduced Contraction time 90% (n = 6; ***P < 0.01), (**c**) reduced Relaxation time 90% (***P < 0.001) at 2 Hz pacing, (**d**) increased maximum capture rate (n = 6; ***P < 0.001), (**e**) shift from a negative to a neutral force-frequency relationship (n = 6; *P < 0.05 to ***P < 0.001 vs. D14), and (**f**) increased cardiomyocyte alignment concentration (κ), [n = 4 (day14) and n = 3 (day28); ***P < 0.001]. (**g**), (**h**) Comparison of gene expression analysis (qPCR) between 14-day and 28-day constructs (n = 3; *P < 0.05 to ***P < 0.001). cTnT level was lower in 28-day constructs, and other genes were normalized to the value of cTnT expression.

We then investigated the impact of prolonged 28-day *in vitro* culture on 6 M ME-ECT maturation. We measured contraction time 90% (from 10% to peak) and relaxation time 90% (from peak to 10%) at 2 Hz electrical pacing (Fig. 4a) and found that 28-day ME-ECT constructs showed significantly more rapid contraction and relaxation compared to 14-day constructs (Fig. 4b,c). Moreover, they captured higher pacing frequency than 14-day constructs (Fig. 4d), with frequency-dependent acceleration of relaxation (Supplementary Figure 4c). Average maximum capture rate increased to 6.9 Hz. We determined active force-frequency relations (FFR) up to 4.5 Hz (270 bpm) and noted that 14-day constructs displayed a progressively negative FFR. On the other hand, although 28-day constructs showed the trend of smaller active stress than 14-day ones at 1.5 Hz, they maintained force even at high beating rate (Fig. 4e and Supplementary Figure 4a,b). The active stress at 2 Hz, 2.5 Hz, and 3 Hz showed the trend of increase from 1.5 Hz (p = 0.067, 0.068, and 0.067 respectively), which can be described as a neutral FFR^28^. According to myofiber alignment analysis, 28-day constructs showed greater alignment than 14-day constructs (Fig. 4f). To further understand the mechanism of functional maturation during prolonged *in vitro* LF-ECT culture, we performed gene expression analysis (qPCR) for selected CM genes relevant to CM structure and function. The expression level of cTnT in 28-day constructs was significantly lower than in 14-day ones [0.53 ± 0.07 versus 1.12 ± 0.16, p = 0.043] (Fig. 4g). We quantified the density of CMs among all cells in cTnT and DAPI stained images of ECTs by counting cTnT^+^ nuclei (not by area fraction) and found a reduced percentage of CMs in 28-day constructs compared to 14-day constructs [30.9 ± 6.3 (n = 4) versus 56.8 ± 10.3% (n = 3), p = 0.11 (Supplementary Figure 5a,b,e)]. Therefore, the lower expression level of cTnT transcripts is consistent with an attrition in the number of CMs with prolonged *in vitro* culture. We examined other genes and normalised their values to cTnT expression level of each group to compare the characteristics of individual CMs in ECTs. 28-day constructs showed a significantly greater ratio of MLC2v and MLC2a, indicating a predominance of maturing ventricular CM (Fig. 4g). We also noted the increase of multiple transcripts consistent with CM maturation including KCNJ2 and KCND3, related to inward rectifier potassium current (I_K1_) and transient outward potassium current (I_to_), respectively^29^. Along with those advanced ion channel functions, increased expression of GJA1 (coding connexin43) is consistent with the rapid contraction and relaxation of the d28 LF-ECTs (Fig. 4h). Interestingly, we noted similar percentages of CD31^+^ ECs in 14-day and 28-day ECTs (31.1 ± 11.3% and 33.0 ± 7.1%, respectively) though there was substantial variance within bundles (Supplementary Figure 5c–e).

**Figure 5:**
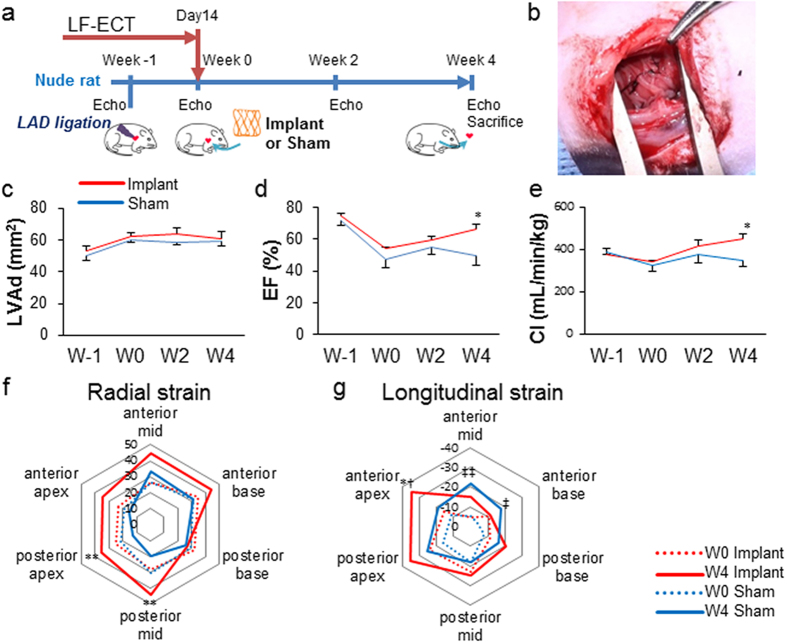
Myocardial functional recovery and regional changes in wall motion after hiPSC-ME-ECTs cardiac implantation. (**a**) Schematic timeline of rat surgery. Echo: echocardiogram. Left anterior descending artery (LAD) ligation is performed (Week -1) then a ME-ECT matured *in vitro* for 14 days (or sham suture) is implanted in a male nude rat (Week 0). Echo is performed prior to LAD ligation on Week-1 (W-1), prior to surgery at Week 0 (W0), then Week 2 (W2) and Week 4 (W4). (**b**) ME-ECT implanted onto the heart surface at infarction site (right). (**c–e**) Results of B-mode echocardiogram [n = 5 (Implant, red) and 5 (Sham, blue)]. (**c**) Left ventricular end diastolic area (LVAd; mm^2^), (**d**) ejection fraction (%), and (**e**) cardiac index, CI (mL/min/kg) [baseline before LAD ligation (W-1), before treatment (W0), and at week 2 (W2) and week 4 (W4), *P < 0.05 Implant versus Sham at W4]. (**f**) Comparison between W0 (dotted line) and W4 (solid line) averaged composite long axis Radial strains for Implant (red, n = 5) and Sham (blue, n = 5). Treatment shows an increase in posterior apex and posterior mid region shortening at W4 after ME-ECT implantation (**P < 0.01 versus W4 Sham). (**g**) Comparison between W0 (dotted line) and W4 (solid line) averaged composite long axis Longitudinal strains for Implant (red, n = 5) and Sham (blue, n = 5). Treatment shows an increase in anterior apex shortening at W4 after ME-ECT implantation (*P < 0.05 versus W4 Sham and ^†^P < 0.05 versus W0 Implant). There is also a compensatory increase in anterior mid (^‡‡^P < 0.01 versus W0 Sham) and anterior base shortening (^‡^P < 0.05 versus W0 Sham) consistent with adaptive remodelling.

To develop the LF-ECT surgical implant method we initially implanted one-half of an ME-ECT onto an uninjured immune tolerant rat heart similar to our approach for linear ECTs^8^,^19^ and performed histology four weeks after the implantation. We confirmed ME-ECT engraftment and vascular coupling between the recipient myocardium and implanted ECT grafts using host venous injection of fluorescent dye-conjugated lectin to identify perfused vessels within the engrafted tissue (Supplementary Figure 6a,b)^8^, recognizing that lectin injection provides evidence of vascular perfusion but is insufficient to determine the extent and function of vessels within the implanted ECT. We then determined the *in vivo* potential of LF-ECT implantation to recover cardiac structure and function following myocardial infarction in an immune tolerant rat MI model (Fig. 5a). A total of 10 rats were randomly divided into two groups: rats implanted with ECTs (n = 5) and sham-operated controls (n = 5). We implanted a whole ECT, folded in half, over the anterolateral left ventricular (LV) surface in the treated group (Fig. 5b). All implanted and sham-operated rats survived the 4-week post-implant observation period with no tumour formation. Although diastolic LV area increased in both groups (Fig. 5c), ECT implantation improved ejection fraction (Fig. 5d), cardiac output (Fig. 5e), and regional LV radial and longitudinal strain at 4 weeks (Fig. 5f,g). We noted both increased radial and longitudinal strain at the infarct region following LF-ECT implantation as well as a compensatory increased anterior mid- and base-longitudinal strain in the non-infarcted regions in sham-operated animals.

**Figure 6:**
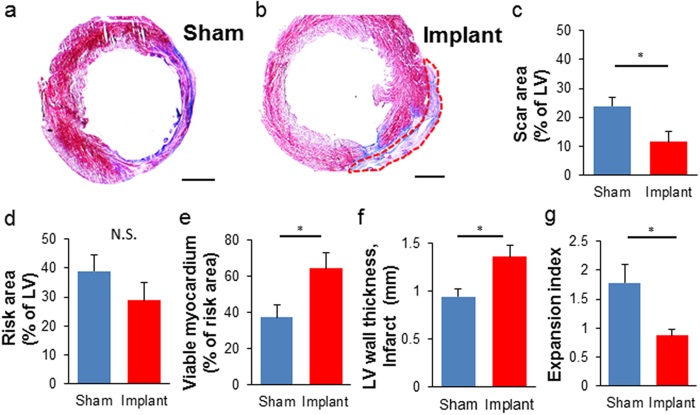
Morphometric analysis of LV remodelling after hiPSC-ME-ECTs cardiac implantation. Representative Masson’s trichrome staining of (**a**) sham treated and (**b**) ME-ECT implanted rat hearts at W4. Scale bar: 2 mm. Red dotted line indicates engrafted area (**c**) Scar area (% of LV), (**d**) Risk area (% of LV), (**e**) Viable myocardium (% of risk area), (**f**) LV wall thickness at the infarct (mm), and (**g**) Expansion index after ME-ECT implantation (*P < 0.05, N.S., not significant). Expansion index = (endocardial circumference/epicardial circumference) × (non-infarcted region wall thickness/risk region wall thickness). Multiple indices support reduced scar and increased viable myocardium after hiPSC-ME-ECT implantation [n = 5 (Implant) and 5 (Sham)].

**Figure 7:**
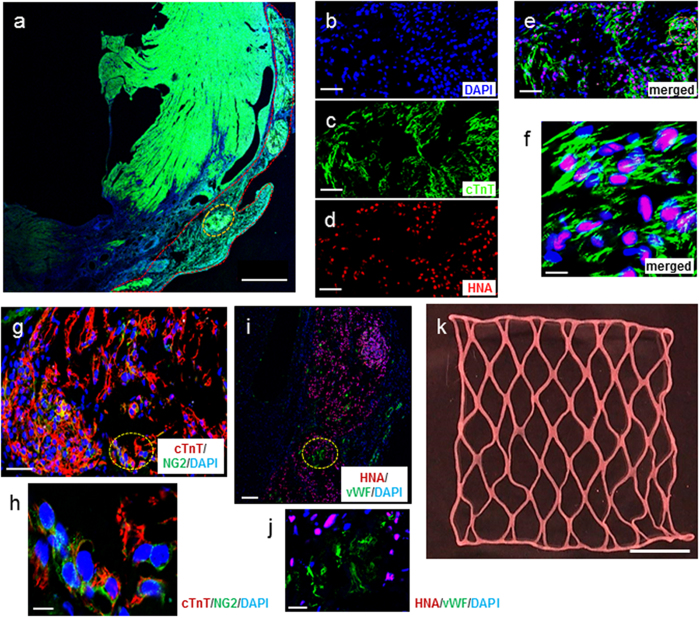
Myocardial regeneration and post-implantation *in vivo* reorganisation of hiPSC- ME-ECT. (**a**) Representative left ventricular histology 4 weeks after implantation of hiPSC-ME-ECT. Scale bar: 1 mm. Red dotted line indicates engrafted area and yellow dotted line represents region seen under higher magnification (**b**–**e**, Scale bar: 50 μm). (**b**) Higher magnification identifying all nuclei via DAPI, nuclear stain; (**c**) lineage-specific stain cTnT; cardiac Troponin T; (**d**) hiPSC-derived cells identified by HNA, human nuclear antigen; (**e**) merged image. Orange dotted line indicates region seen under higher magnification in (**f**) triple immunostaining showing sarcomeric structure in hiPSC-derived cardiomyocytes. Scale bar: 10 μm. (**g**) Colocation of CM and mural cells. All nuclei, DAPI; mural cells, NG2 proteoglycan; CM, cTnT. Some cells stained positive for both cTnT and NG2. Scale bar: 50 μm. Yellow dotted line indicates region seen under higher magnification in in (**h**, Scale bar: 10 μm). (**i**) Triple immunostaining showing hiPSC-derived cells by HNA, endothelial cells identified by von Willebrand factor, vWF, and nuclei by DAPI. Scale bar: 100 μm. Yellow dotted line indicates region seen under higher magnification in (**j**, Scale bar: 20 μm). (**k**) Representative images of extra large-format ME-ECT intended for large animal preclinical trials after release from the PL7 mold. Scale bar: 10 mm.

Consistent with functional recovery, LF-ECT implantation reduced histologic measures of scar size (Fig. 6a–c) with a trend to reduce risk area (Fig. 6d), and increased viable myocardium (Fig. 6e) and mean LV wall thickness in the risk area (Fig. 6f). Finally, the lower expansion index in implanted rats indicated reduced post-MI LV remodelling (Fig. 6g)^30^. All implanted rats exhibited ECT engraftment (n = 5), and we confirmed the retention of grafted hiPSC-derived cTnT^+^ CMs with sarcomeric structures (Fig. 7a–f) and NG2^+^ mural cells (Fig. 7g,h). Interestingly we noted a small number of hiPSC-derived cells that costained for cTnT and NG2, consistent with the expression of NG2 by immature cardiomyocytes^31^ or the less likely expression of cTnT by human cardiac pericytes^32^. In addition, we confirmed the distribution of vWF^+^ endothelial cells (Fig. 7i,j) inside the ECT. Most vWF^+^ endothelial cells were HNA negative, indicating the invasion of host-derived vasculature into the graft. We did not note LF-ECT derived, HNA^+^ beyond the margins of the implanted LF-ECTs.

Finally, as a proof of feasibility for scaling up the LF-ECT method for large animal pre-clinical studies, we generated a 3 cm final width extra-large format ME-ECTs (XLF-ECT) from a 4 cm square wide mold based on the PL7 design and containing 24 million cells (Fig. 7k). Similar to the original ME-ECTs, we could easily collect the spontaneously beating XLF-ECTs from the mold without damaging the ECT. According to the force measurement analysis (n = 3), electromechanical parameters of XLF-ECTs including active stress (0.61 ± 0.08 mN/mm^2^ at 2 Hz/5 V pacing), maximum capture rate (5.2 ± 0.7 Hz), excitation threshold voltage (1.7 ± 0.2 V) were similar to the smaller LF-ECTs. These results indicate that the geometry and size of ME-ECTs can be adjusted to generate larger ECTs towards preclinical larger animal trials.

## Discussion

Multiple formulations (cell species, lineage, matrix composition, geometry) of ECTs have been reported, and these microtissues are suitable for *in vitro* drug toxicity screening and disease modelling^15^,^33^. Several have been used as implantable grafts in rodents^8^,^13^,^16^,^19^, though they are too small for larger animal trials or clinical translation. In the present study, we describe the successful incorporation, survival, and functional maturation of hiPSC-derived cardiac and vascular cells in a scalable, porous LF-ECT suitable for *in vivo* translation.

In accordance with the results of our previous study^8^, we generated ECTs from hiPSC-derived CMs, ECs, and MCs mixed at about 3:1:1 ratio as shown in Supplementary Figure 1b. Meanwhile, there were some remaining cell populations including undifferentiated hiPSCs. It is important to establish a method to eliminate undifferentiated cells considering clinical application of hiPSC-ECTs in the future. Although other cell components have not been fully characterized yet, we have determined the percentage of the additional markers: CD44 and CD90 (Thy1), which are known as classical mesenchymal stem cell and fibroblast markers, respectively.

Several technical aspects of our approach are worth noting. We used thin PDMS sheets to design the 3D molds used to generate LF-ECTs, and once coated with Pluronic F127, the LF-ECTs remained detached from the adjacent posts and the bottom during *in vitro* maturation, facilitating gel compaction and final removal from the mold. Comparison of 3 distinct geometries identified the PL7 ME-ECT as displaying preferred structural and functional properties. PL0 PS-ECTs had higher cell death *in vitro*. They also showed reduced cell alignment and lower active force compared to other structures, which indicates poorer mechanical loading and suggests a less optimal pre-implant construct.

The PL16 ML-ECT mold had the longest posts but produced ECTs with variable bundle widths, most likely due to uneven cell distribution during gel pouring or to variations in mechanical loading from the ends of these longer bundles versus the shorter PL7 ME-ECT bundles. Interestingly, ML-ECTs also showed a higher percentage of dead cells than ME-ECTs, perhaps related to the irregular shapes and loading of bundles. More cell death could happen due to variable mechanical stresses concentration along the long ML-ECT bundles. Another explanation related to bundle nutrition could be that the bundles in ME-ECTs are suspended between junctions at both ends and almost completely detached from the bottom, optimising media delivery of nutrition. On the other hand, we noted that some of the bundles in ML-ECTs touched the bottom, which might reduce nutrient delivery by restricting the medium beneath the construct. PL7 ME-ECTs had reproducibly uniform bundle widths and increased cell alignment as well as structural durability during *in vitro* handling and *in vivo* implantation. Hence, we consider ME-ECTs to have preferred scalability and reproducibility compared to ML-ECTs.

In addition to active stress, we measured maximum capture rate and excitation threshold voltage during force measurement analysis as an index of CM electrophysiological maturation. There was no difference among three geometries, and each value was comparable to CM + EC + MC type linear ECTs in our previous study^8^. Thus, those parameters were not as affected by tissue geometry as by the cellular composition^8^ or duration of *in vitro* maturation.

Next, with the aim of delivering more cardiac cells and generating thicker constructs, we tried to increase the initial seeding cell number at the same or doubled cell density per construct using the ME-ECT format. By increasing the initial cell number up to 12 M keeping the standard density, we succeeded in forming thicker constructs. However, that worsened cell viability probably due to the depletion of nutrition and oxygen inside constructs. We then reduced the initial volume of 12 M ECTs to half and generated the fourth group of 12 MH ECTs, which formed thinner bundles than 12 M ECTs. However, the dead cell ratio of the group was similar to 12 M and more than four times greater than that of 6 M, suggesting that nutrient delivery alone does not explain the differences in cell survival. In conclusion, the original 6 M cells showed the best cell survival and ECT function, indicating a cell number and density limit with this formulation.

In our previous study, inclusion of ECs and MCs in linear hiPSC ECTs resulted in more prominent sarcomeric structural maturation^8^. Here we investigated the impact of prolonging *in vitro* 3D culture to 4 weeks on the electromechanical maturation of ME-ECTs. Although 28-day constructs tended to reduce active stress at 1.5 Hz pacing versus 14-day constructs, they showed the change of force-frequency relationship (FFR) from negative to neutral^28^. A positive FFR, termed the Bowditch effect, is a functionally important feature of mature myocardium and allows the cardiac pump to adapt to the continually altering hemodynamic needs of the body^34^. The shift from a negative to a neutral FFR in 28-day constructs represents continued functional maturation as well as more rapid force generation and relaxation cycle, and the ability of capturing higher pacing frequency. Despite increased CM maturation, CM content decreased in 28-day constructs compared to 14-day constructs (Supplementary Figure 5e), suggesting that a prolonged *in vitro* environment is likely less physiologic than *in vivo.* On the other hand, CD31 staining showed the survival and expansion of ECs from the initial 15.9% by flow cytometry to over 30% after 14-day culture, which was maintained until day 28. Furthermore, extended *in vitro* culture beyond 28 days was associated with migration of stromal-like cells from the floating LF-ECTs to the construct mold, which resulted in some tethering of the construct. Therefore, due to the continued maturation of CM within the 28-day culture LF-ECTs, longer culture times may be preferable for applications that use hiPSC-CM for drug screening and disease modeling.

Our ultimate goal is translation of the LF-ECT paradigm towards clinical therapies and therefore validation of additional efficacy in *in vivo* models is required for each modification of the ECT paradigm. In an immune tolerant xenograft rat model, we confirmed *in vivo* survival of LF-ECT grafted cells on both non-MI and MI hearts. The invasion of host-derived vasculature into the graft and vascular integration between the host and the graft was confirmed histologically. LF-ECT implantation attenuated ventricular remodelling and recovered cardiac function after myocardial infarction. Importantly, LF-ECT contributed to the preservation of viable myocardium at the risk region leading to the maintenance of wall thickness and the prevention of scar formation. According to regional strain analysis, the regional wall motion at and around the risk region recovered significantly compared to the sham operated controls. Thus, survival of implanted hiPSC-derived ECTs was confirmed at four-week follow-up and was associated with both structural and functional myocardial recovery, though the mechanisms responsible for this recovery are yet to be identified.

In conclusion, we generated LF-ECTs from hiPSC-derived cardiac cells using PDMS tissue molds coated with Pluronic F127. LF-ECT geometry alters cell survival, tissue maturation, and function, identifying PL7 ME-ECT as the preferred geometry. LF-ECTs cell composition also alters survival, maturation, and function, identifying 6 M as optimal for cell survival and force production with continued maturation of CM performance with extended *in vitro* 3D culture to 28 days. LF-ECTs survived *in vivo* and improved cardiac function after MI. We can expand the scale of 15 × 15 mm LF-ECTs to larger formats such as 30 × 30 mm XLF-ECT for large animal trials, including exploring ECT stacking as has been accomplished with cell sheets. Scalable large-format ECTs may be feasible for hiPSC-based preclinical and clinical cardiac regeneration paradigms.

## Materials and Methods

Detailed methods are described in Supplementary Methods.

### Human iPSC Culture and Differentiation

Human iPSCs [4-factor (Oct3/4, Sox2, Klf4 and c-Myc) line: 201B6 and culture methods were used as previously described^8^,^10^. In brief, these cells were adapted and maintained on thin-coat Matrigel (growth factor reduced, 1:60 dilution; BD Biosciences, San Jose, USA) in mouse embryonic fibroblast conditioned medium (MEF-CM) supplemented with 4 ng/mL human basic fibroblast growth factor (hbFGF; WAKO, Osaka, Japan).

Cardiovascular (CV) cell differentiation was induced as previously reported (Supplementary Figure 1a)^4^,^8^,^35^. Cells were detached following a Versene (0.48 mM EDTA solution; Life Technologies, Carlsbad, USA) treatment and seeded onto Matrigel-coated plates at a density of 1,000 cells/mm^2^ in MEF-CM with 4 ng/mL bFGF for 2 to 3 days before induction. Cells were covered with Matrigel (1:60 dilution) on the day before induction. To induce CV cell population, we replaced MEF-CM with RPMI + B27 medium (RPMI1640; Life Technologies, 2 mM L-glutamine; Life Technologies, 1× B27 supplement without insulin; Life Technologies) supplemented with 100 ng/mL of Activin A (R&D, Minneapolis, USA) and 100 ng/mL of Wnt3a (R&D) for 24 hours (differentiation day 0; d0), followed by 10 ng/mL human bone morphogenetic protein 4 (BMP4; R&D) and 10 ng/mL hbFGF (d1) for 2 or 4 days without culture medium change. For induction of CM and EC (CM + EC protocol): The culture medium was replaced at d5 with RPMI + B27 supplemented with VEGF165 (Miltenyi, Bergisch Gladbach, Germany), and culture medium was refreshed every other day. Beating cells appeared at d11 to 13. For induction of MC (MC protocol): The culture medium was replaced at d3 with RPMI + 10% FBS medium [RPMI1640, 2 mM L-glutamine, 10% fetal bovine serum (FBS)], and culture medium was refreshed every other day (Supplementary Figure 1a).

### Tissue Mold Fabrication

Tissue molds were fabricated from Polydimethylsiloxane (PDMS) (Fig. 1b)^25^. Thin (0.5 mm) layer of PDMS (Sylgard 184, Dow Corning) was cast by mixing the prepolymer and cross-linking solution at a ratio of 10:1 and allowed to cure at 80 °C for three hours. The sheet was cut and bonded with silicone adhesive to fabricate tissue trays according to design drawings. Tissue molds were 21 mm × 20.5 mm in outer diameter and had 0.5 mm wide and 2.5 mm high PDMS rectangular posts with three different patterns: PL7, PL16, and PL0 (Fig. 1c). PL7 had 7 mm long posts at a staggered position and PL16 had parallel 16 mm long posts. PL0 has 1 mm long pins at the periphery and no posts in the middle. Horizontal post spacing between two lines of posts was 2.5 mm (Fig. 1b). For the generation of an extra-large format ECT (XLF-ECT, Fig. 7k), a 39 mm × 40.5 mm mold with PL7 patterned posts was also fabricated. Molds were autoclaved and coated with 1% Pluronic F127 [Pluronic^®^ F-127 10% Solution (Molecular Probes) diluted with PBS] for one hour. Before tissue moulding, Pluronic F127 was removed, and the mold was rinsed with PBS sufficiently.

### LF-ECT Fabrication

We combined differentiated cells from CM + EC and MC protocols so that the final concentration of MCs became 10 to 20%. Combined cells were mixed with acid-soluble rat-tail collagen type I (Sigma) and matrix factors (Matrigel; BD Biosciences). Cell/matrix mixture was performed as follows^8^,^18^,^19^. For a 6 M construct fabrication: (1) Six million cells were suspended in a culture medium (high glucose-modified Dulbecco’s essential medium; Life Technologies) containing 20% fetal bovine serum (Life Technologies). (2) Acid-soluble collagen type I solution (pH 3) was neutralised with alkali buffer (0.2 M NaHCO3, 0.2 M HEPES, and 0.1 M NaOH) on ice. (3) Matrigel (15% of total volume) was added to the neutralised collagen solution. (4) Cell suspension and matrix solution were mixed. The final concentration of collagen type I was 0.67 mg/mL in a total volume of 400 μL (Fig. 1a).

The cell/matrix mixture was poured onto the Pluronic F127 coated PDMS tissue mold, which was placed in a usual six-well culture plate and polymerised in a standard CO_2_ incubator (37 °C, 5% CO_2_) for 60 minutes. When the tissue was formed, the tissue mold was soaked with pre-culture medium [alpha minimum essential medium (αMEM; Life Technologies) supplemented with 10% FBS, 5 × 10-5 M 2-mercaptoethanol (Sigma) and 100 U/mL Penicillin-Streptomycin (Life Technologies)]. Constructed ECTs were cultured for 14 days with medium change every day and analysed at day14. A gel solution of 9 million cells, 600 μL or 12 million cells, 800 μL was prepared for the fabrication of 9 M or 12 M construct. Furthermore, a 12 million cell, 400 μL cell/matrix mixture, which has two times higher cell density than others, was prepared for 12 MH construct (Fig. 3a). For an XLF-ECT, a cell/matrix mixture of 24 million cells in 1600 μL matrix was used.

### Live/Dead Assay

ECTs were incubated with staining solution (50 mL/L Ethidium Homodimer III and 50 mL/L Hoechst 33342, PromoCell GmbH, Heidelberg, Germany) in a pH-adjusted buffer for 60 minutes at room temperature and protected from light^22^. Fluorescent images were obtained using an Olympus DP72 optical microscope (Olympus, Tokyo, Japan).

### Contractile Force Measurement

For contractile force measurements, a strip with the length of approximately 15 mm was cut off from an ECT (Supplementary Figure 3). As previously described^8^,^18^,^19^ the strip was preserved in cold (25 °C) Tyrode’s solution containing (in mM) 119.8 NaCl, 5.4 KCl, 2.5 CaCl_2_, 1.05 MgCl_2_, 22.6 NaHCO_3_, 0.42 NaH_2_PO_4_, 0.05 Na_2_EDTA, 0.28 ascorbic acid, 5.0 glucose, and 30 2,3-butanedione monoxime (BDM) gassed with 95% O_2_ and 5% CO_2_. One end of the specimen was gently attached to a force transducer (model 403 A, Aurora Scientific, Ontario, Canada) and the other end to a high-speed length controller (model 322 C, Aurora Scientific) mounted on a micromanipulator using 10-0 nylon threads. The perfusion chamber containing the construct was then filled with BDM-free warmed Tyrode’s solution (37 °C, 1 ml total volume). During a 20 minute equilibration period, the construct was field-stimulated (2 Hz/5 V). The segment length of the tissue was gradually increased until total force reached maximum (*Lmax*). According to the 3D reconstructed confocal images of whole tissues, each bundle of an ME or ML-ECT could be assumed to have an elliptical cylindrical shape. The minor to major axis ratio of the representative cross-section was 0.65 in average, and there was no significant difference among all groups. We estimated the cross-sectional area (CSA, mm^2^) from the mean bundle width while strained on the tissue mold and the axis ratio. We measured 1) active force under 1.5–4.5 Hz and 5 V pacing at slack length to *Lmax*, 2) maximum capture rate (without capturing failure for 10 seconds) under 5 V at *Lmax*. Active stress (mN/mm^2^) was calculated by dividing the value of active force by CSA.

### Cardiomyocyte Alignment Analysis

We compared CM alignment among the ECT groups based on confocal images of cTNT immunostained samples. Three-dimensional (3D) confocal stacks of whole-mount ECT bundles were acquired at 10X magnification and 3 μm spacing (Fig. 3c). Local CM orientations were computed from the image gradient and transformed to cylindrical coordinates based on the ECT bundle centerline. The CM alignment was then defined as the concentration parameter (κ) for the associated Watson distribution of all local orientations. A larger к value indicates that CM orientations are more concentrated along a single direction. The analysis was carried out in Matlab (MathWorks, Natick, MA).

### Animal Model Preparation and ECT Implantation

All animal surgeries were performed following protocols approved by the University of Louisville Institutional Animal Care and the *Guide for the Care and Use of Laboratory Animals* prepared by the Institute for Laboratory Animal Research, USA (8th ed., 2011). Male athymic nude rats (NTac: NIH-Foxn1^^rnu^^, Taconic Biosciences, Hudson, USA) weighing 270–340 g were used as recipients for surgeries.

One-half of ME-ECT was implanted in a normal nude rat to confirm the engraftment of the ECT on a rat heart through left thoracotomy. We then implanted an ME-ECT in a myocardial infarction (MI) model rat (Fig. 5a). MI was induced by permanent left anterior descending artery ligation using 7-0 silk suture as previously described^8^,^19^. Isoflurane (3–5%) inhalation was used for general anaesthesia, and subcutaneous or intraperitoneal injection of Buprenorphine (0.5 mg/kg, twice a day, three days including operation day) was used for analgesia. ECT implantation was performed one week after MI induction during the “subacute phase” of MI. Left ventricular ejection fraction (EF) was evaluated six days after coronary artery ligation by echocardiography. Rats, whose hearts showed more than 60% EF or preserved more than 80% of initial EF, were excluded from subsequent experiments. A total of 10 rats were randomly divided into two groups: rats implanted with ECTs (Implant group; n = 5) and sham-operated rats (Sham group; n = 5). As previously described, the LV anterior wall was exposed through redo-left thoracotomy^8^,^19^. Using 7-0 silk sutures, the anterior infarcted myocardium was covered with one whole ME-ECT, folded in half, along the LV circumferential direction. For the sham-operated group, a thoracotomy was performed one week after coronary ligation; however, no ECT implantation was performed. Hearts were harvested 4 weeks after treatment or sham operation and prepared for Masson’s trichrome and immunohistochemistry (Fig. 5a).

### Cardiac Functional Assessment

Transthoracic echocardiography was performed by an investigator blinded to group assignment using a high resolution Vevo2100 system (VisualSonics, Toronto, Canada) and 21-MHz imaging transducer (MS250; VisualSonics). Evaluations were performed before MI, ECT implantation (six days after MI induction), and two and four weeks after implantation (Fig. 5a). Stroke volume (SV) and ejection fraction was calculated by single plane area-length method. Cardiac index (CI) was calculated by the following formula: CI = SV × heart rate/body weight. Regional wall motion was traced, calculated and visualised using the VevoStrain application (VisualSonics)^36^.

### Histologic Measurement of Left Ventricular Remodeling

To quantify the LV remodelling after MI with or without ECT implantation, five 8 μm frozen sections with 50 μm interval were stained with Masson’s trichrome. Sections were imaged under Olympus DP72 optical microscope (Olympus, Tokyo, Japan) and analysed using NIH ImageJ. Just like Tang’s report^30^, a series of morphometric parameters were measured in each section including total LV area, scar area, risk region area, LV wall thickness in the risk and non-infarcted regions, and LV expansion index. Risk area was defined as the LV area between the two edges of the infarct scar. Wall thickness was the average of 5 measurements equally distributed within risk and non-risk area. The LV expansion index was calculated from LV circumference and wall thickness to evaluate both LV dilation and wall thinning simultaneously: Expansion Index = (endocardial circumference/epicardial circumference) × (noninfarcted region wall thickness/risk region wall thickness).

### Statistical Analysis

The data were analysed using JMP software for Windows (version10.0.2, SAS Institute Inc., Cary, NC). Results are presented as mean ± standard error of the mean (SEM). Comparisons between two groups were made with the unpaired t-test unless otherwise noted. Mann-Whitney U test was used for non-normal distributions. Comparisons between more than 2 groups were made with one-way or two-way repeated analysis of variance (ANOVA) followed by Tukey’s test as post hoc. A p-value of less than 0.05 was considered significant.

## Additional Information

**How to cite this article**: Nakane, T. *et al*. Impact of Cell Composition and Geometry on Human Induced Pluripotent Stem Cells-Derived Engineered Cardiac Tissue. *Sci. Rep.* **7**, 45641; doi: 10.1038/srep45641 (2017).

**Publisher's note:** Springer Nature remains neutral with regard to jurisdictional claims in published maps and institutional affiliations.

## Supplementary Material

Supplementary Information

Supplementary Video 1
