# Supplementary material for: Impact of Cell Composition and Geometry on Human Induced Pluripotent Stem Cells-Derived Engineered Cardiac Tissue
Source: Sci Rep. 2017 Apr 3;7:45641. doi: 10.1038/srep45641 (PMC5377302; doi:10.1038/srep45641)
Supplement: Supplementary Information [file srep45641-s1.pdf]

Supplementary Information

Impact of Cell Composition and Geometry on Human Induced Pluripotent Stem Cells-Derived Engineered Cardiac Tissue

Takeichiro Nakane[1,2,3], Hidetoshi Masumoto[1,2,3], Joseph P. Tinney[1,4], Fangping Yuan[1,4], William J. Kowalski[1,4], Fei Ye[1,4], Amanda J. LeBlanc[5], Ryuzo Sakata[3], Jun K. Yamashita[2], Bradley B. Keller[1,4*]

[1] Kosair Charities Pediatric Heart Research Program, Cardiovascular Innovation Institute, University of Louisville, Louisville, Kentucky, The United States of America

[2] Department of Cell Growth and Differentiation, Center for iPS Cell Research and Application (CiRA), Kyoto University, Kyoto, Japan

[3] Department of Cardiovascular Surgery, Kyoto University Graduate School of Medicine, Kyoto, Japan

[4] Department of Pediatrics, University of Louisville School of Medicine, Louisville, Kentucky, The United States of America

[5] Department of Physiology, University of Louisville, Louisville, Kentucky, The United States of

*Corresponding author:
Bradley B. Keller, M.D.
Kosair Charities Endowed Chair and Director, Pediatric Heart Research Program, Cardiovascular Innovation Institute, University of Louisville
302 East Muhammad Ali Blvd, Louisville, Kentucky 40202, The United States of America
TEL: +1-502-852-5809, FAX: +1-502-852-7195
E-mail: brad.keller@louisville.edu
URL: http://cv2i.org

Supplementary Methods

Human iPSC Culture and Differentiation

Human iPSCs [4-factor (Oct3/4, Sox2, Klf4 and c-Myc) line: 201B6 and culture methods were used as previously described[1,2]. In brief, these cells were adapted and maintained on thin-coat Matrigel (growth factor reduced, 1:60 dilution; BD Biosciences, San Jose, USA) in mouse embryonic fibroblast conditioned medium (MEF-CM) supplemented with 4 ng/mL human basic fibroblast growth factor (hbFGF; WAKO, Osaka, Japan). Cells were passaged every four or five days using CTK solution [0.1% collagenase IV, 0.25% Trypsin, 20% knockout serum replacement (KSR), and 1 mM CaCl2 in phosphate buffered saline (PBS)]. Cardiovascular (CV) cell differentiation was induced as previously reported (Supplementary Fig. 1a)[1,3,4]. Cells were detached following 4 to 7 minutes incubation with Versene (0.48mM EDTA solution; Life Technologies, Carlsbad, USA) and seeded onto Matrigel-coated plates at a density of 1,000 cells/mm^2 in MEF-CM with 4 ng/mL bFGF for 2 to 3 days before induction. Cells were covered with Matrigel (1:60 dilution) on the day before induction. To induce CV cell population, we replaced MEF-CM with RPMI+B27 medium (RPMI1640; Life Technologies, 2mM L-glutamine; Life Technologies, 1× B27 supplement without insulin; Life Technologies) supplemented with 100 ng/mL of Activin A (R&D, Minneapolis, USA) and 100 ng/mL of Wnt3a (R&D) for 24 hours (differentiation day 0; d0), followed by 10 ng/mL human bone morphogenetic protein 4 (BMP4; R&D) and 10 ng/mL hbFGF (d1) for 2 or 4 days without culture medium change. For induction of CM and EC (CM+EC protocol): The culture medium was replaced at d5 with RPMI+B27 supplemented with VEGF165 (Miltenyi, Bergisch Gladbach, Germany), and culture medium was refreshed every other day. Beating cells appeared at d11 to 13. For induction of MC (MC protocol): The culture medium was replaced at d3 with RPMI+10% FBS medium [RPMI1640, 2mM L-glutamine, 10% fetal bovine serum (FBS)], and culture medium was refreshed every other day (Supplementary Fig. 1).

Flow Cytometry

HiPSC-derived CV cells were dissociated by incubation with Accumax (Innovative Cell Technologies, San Diego, CA) and stained with one or a combination of the following surface markers: anti-VCAM1 conjugated with allophycocyanin (APC), clone STA, 1:200 (BioLegend, San Diego, CA); anti-PDGFRβ conjugated with phycoerythrin (PE), clone 28d4, 1:100 (BD, Franklin Lakes, NJ); anti-VE-cadherin conjugated with fluorescein isothiocyanate (FITC), clone 55-7h1, 1:100 (BD); anti-TRA-1-60 conjugated with FITC, clone TRA-1-60, 1:20 (BD). Anti-CD44 conjugated with FITC, clone BJ18, 1:100 (BioLegend), and anti-CD90 (Thy1), clone 5E10 (BioLegend) labelled with Alexa-488 using Zenon technology (Life Technologies) (1:50) was used for additional analysis. To eliminate dead cells, cells were stained with the LIVE/DEAD fixable Aqua dead cell staining kit (Life Technologies). For cell surface markers, staining was carried out in PBS with 5% FBS. For intracellular proteins, staining was carried out on cells fixed with 4% paraformaldehyde (PFA) in PBS. Cells were stained with the anti-cardiac isoform of Troponin T (cTnT) (clone 13211, Thermo Fisher Scientific, Waltham, MA) labelled with Alexa-488 using Zenon technology (1:50). The staining was performed in PBS with 5% FBS and 0.75% Saponin (Sigma, St. Louis, MO). Stained cells were analyzed on

an LSRII flow cytometer (BD). Data were collected from at least 5,000 events. Data were analyzed with DIVA software version 6.1.3 (BD).

Tissue Mold Fabrication

Tissue molds were fabricated from polydimethylsiloxane (PDMS) (Fig. 1b)[5]. Thin (0.5 mm) layer of PDMS (Sylgard 184, Dow Corning) was cast by mixing the prepolymer and cross-linking solution at a ratio of 10:1 and allowed to cure at 80°C for three hours. The sheet was cut and bonded with silicone adhesive to fabricate tissue trays according to design drawings. Tissue molds were 21 mm x 20.5 mm in outer diameter and had 0.5 mm wide and 2.5 mm high PDMS rectangular posts with three different patterns: PL7, PL16, and PL0 (Fig. 1c). PL7 had 7 mm long posts at a staggered position and PL16 had parallel 16 mm long posts. PL0 has 1 mm long pins at the periphery and no posts in the middle. Horizontal post spacing between two lines of posts was 2.5 mm (Fig. 1b). For the generation of an extra-large format ECT (XLF-ECT, Fig. 7k), a 39 mm x 40.5 mm mold with PL7 patterned posts was also fabricated. Molds were autoclaved and coated with 1% Pluronic F127 [Pluronic® F-127 10% Solution (Molecular Probes) diluted with PBS] for one hour. Before tissue moulding, Pluronic F127 was removed, and the mold was rinsed with PBS sufficiently.

LF-ECT Fabrication

We combined differentiated cells from CM+EC and MC protocols so that the final concentration of MCs became 10 to 20%. Combined cells were mixed with acid-soluble rat-tail collagen type I (Sigma) and matrix factors (Matrigel; BD Biosciences). Cell/matrix mixture was performed as follows[1,6,7]. For a 6M construct fabrication: (1) Six million cells were suspended in a culture medium (high glucose-modified Dulbecco's essential medium; Life Technologies) containing 20% fetal bovine serum (Life Technologies). (2) Acid-soluble collagen type I solution (pH 3) was neutralised with alkali buffer (0.2M NaHCO3, 0.2M HEPES, and 0.1M NaOH) on ice. (3) Matrigel (15% of total volume) was added to the neutralised collagen solution. (4) Cell suspension and matrix solution were mixed. The final concentration of collagen type I was 0.67 mg/mL in a total volume of 400 μL (Fig. 1a).

The cell/matrix mixture was poured onto the Pluronic F127 coated PDMS tissue mold, which was placed in a usual six-well culture plate, and polymerised in a standard CO_2 incubator (37° C, 5% CO_2) for 60 minutes. When the tissue was formed, the tissue mold was soaked with pre-culture medium [alpha minimum essential medium (αMEM; Life Technologies) supplemented with 10% FBS, 5×10-5M 2-mercaptoethanol (Sigma) and 100U/mL Penicillin-Streptomycin (Life Technologies)]. Constructed ECTs were cultured for 14 days with medium change every day and analysed at day14. A gel solution of 9 million cells, 600 μL or 12 million cells, 800 μL was prepared for the fabrication of 9M or 12M construct. Furthermore, a 12 million cell, 400 μL cell/matrix mixture, which has two times higher cell density than others, was prepared for 12MH construct (Fig. 3a). For an XLF-ECT, a cell/matrix mixture of 24 million cells in 1600 μL matrix was used.

Live/Dead Assay

ECTs were incubated with staining solution (50 mL/L Ethidium Homodimer III and 50 mL/L Hoechst 33342, PromoCell GmbH, Heidelberg, Germany) in a pH-adjusted buffer for 60 minutes at room temperature and protected from light[8]. Fluorescent images were obtained using an Olympus DP72 optical microscope (Olympus, Tokyo, Japan).

Contractile Force Measurement

For contractile force measurements, a strip with the length of approximately 15mm was cut off from an ECT (Supplementary Fig. 3), comparable to the length of linear ECTs. As previously described[1,6,7] the strip was preserved in cold (25°C) Tyrode solution containing (in mM) 119.8 NaCl, 5.4 KCl, 2.5 $CaCl_2$, 1.05 $MgCl_2$, 22.6 $NaHCO_3$, 0.42 NaH_2PO_4, 0.05 Na_2EDTA, 0.28 ascorbic acid, 5.0 glucose, and 30 2,3-butanedione monoxime (BDM) gassed with 95% O_2 and 5% CO_2. One end of the specimen was gently attached to a force transducer (model 403A, Aurora Scientific, Ontario, Canada) and the other end to a high-speed length controller (model 322C, Aurora Scientific) mounted on a micromanipulator using 10-0 nylon threads. The perfusion chamber containing the construct was then filled with BDM-free warmed Tyrode solution (37°C, 1 ml total volume). During a 20 minute equilibration period, the construct was field-stimulated (2Hz / 5V). The segment length of the tissue was gradually increased until total force reached maximum (*Lmax*). According to the 3D reconstructed confocal images of whole tissues, each bundle of an ME or ML-ECT could be assumed to have an elliptical cylindrical shape. The minor to major axis ratio of the representative cross-section was 0.65 in average, and there was no significant difference among all groups. We estimated the cross-sectional area (CSA, mm^2) from the mean bundle width while strained on the tissue mold and the axis ratio. We measured 1) active force under 1.5-4.5 Hz and 5 V pacing at slack length to *Lmax*, 2) maximum capture rate (without capturing failure for 10 seconds) under 5 V at *Lmax*. Active stress (mN/mm^2) was calculated by dividing the value of active force by CSA.

Histology

For whole mount tissue staining, ECTs were fixed in 4% PFA for 30 minutes at room temperature (RT) and washed in 1% Triton-X-100/PBS for 1 hour at RT, followed by blocking with 1% Triton-X-100/PBS +10% FBS for 1 hour at RT. ECTs were incubated with primary antibody Troponin T (mouse monoclonal; clone 13-11,MS-295, Thermo Fisher Scientific; 1:400) in 1% Triton-X-100/PBS+ 10% FBS+0.02% sodium azide overnight at 4°C at the shaker. On the second day, after washed with 1% Triton-X-100/PBS+ 10% FBS, ECTs were incubated with donkey anti-mouse IgG secondary antibody with Alexa Fluor 488 conjugated overnight at 4°C. After stained with DAPI for 30 minutes, ECTs were incubated with 100% glycerol overnight, 75% glycerol 2 hours, clearing solution 2 hours and changed to new clearing solution overnight. All the clearing procedures were performed at room temperature in the dark. ECTs also were fixed in 4% PFA for 30 minutes at room temperature (RT) and processed to paraffin blocks to cut 5- μm paraffin section for IF staining.

Hearts were arrested in diastole using 200 μl of chilled 3M KCl and 0.1 M CdCl solution followed by 4% PFA (perfusion fixation), embedded in OCT compound (Sakura Finetek Japan, Tokyo, Japan) and frozen. Sequential sections from hearts (8 μm thickness) were prepared for Masson Trichrome staining for scar and for

cell-specific antibody staining. For immunofluorescence staining, sections were treated with 0.01 mol/L sodium citrate buffer (pH 6.0) at 100°C for 15 minutes and incubated with primary antibodies overnight at 4°C; Alexa Fluor 488/594 conjugated donkey anti-mouse, donkey anti-rabbit and donkey anti-rat (1:100 to 1:400, Life Technologies) were used as secondary antibodies. Engrafted human cells in ECTs were detected by human nuclear antibody (HNA) (mouse monoclonal, clone 235-1; Millipore, Billerica, MA; 1:300). Anti-cTnT antibody (ab45932, rabbit polyclonal; Abcam, Cambridge, UK; 1:400) was used for double staining with cTnT and HNA. The following antibodies were used for primary antibodies: anti-von Willebrand factor (vWF) (DAKO, Carpinteria, CA; 1:50); anti-CD31 (rabbit polyclonal; Abcam, Cambridge, UK; 1:20), ant-NG2 (rabbit polyclonal; Abcam, Cambridge, UK; 1:100). Nuclei were visualised with DAPI (4, 6 diamidino -2-phenylindole; Life Technologies). For visualisation of perfused vasculature within ECTs after implantation, 10 μg/g of DyLight 488 Labelled Lycopersicon Esculentum (Tomato) Lectin (Vector Laboratories, Burlingame, CA) was administrated from right jugular vein under general anaesthesia and mandatory respiration. After 20 min of systemic perfusion, the rat was euthanised. The heart was freshly harvested and embedded in OCT compound and frozen for sectioning. All immunostained sections were photographed with Nikon ECLIPSE Ti Confocal System (Nikon, Tokyo, Japan) attached to a Nikon Ti-E inverted microscope platform; images were captured at 1024×1024 pixel density with 1.4 NA using Nikon NIS Elements AR software (Nikon) to save as 12-bit raw files for further processing.

Cardiomyocyte Alignment Analysis

Alignment of CM in ECT bundles was quantified from 3D confocal stacks of whole-mount cTnT immunostained samples. For each ECT group (ME, PL, PS, 6M ME, 9M ME, 12M ME, 12MH ME), we analysed 4 to 6 images, where the mean image size was 1.3 x 1.7 x 0.45 mm. We applied attenuation correction[9] followed by a low pass Gaussian filter (standard deviation of 3 μm) prior to any analysis (ImageJ, NIH, Bethesda, MD). We merged the cTnT and DAPI fields in to a single grayscale image and manually segmented the whole ECT bundle with Seg3D (Salt Lake City, UT)[10]. The centerline (medial axis) of the ECT bundle was extracted from the distance transform of the segmented image[11] and used to form an ECT-based coordinate system, similar to cylindrical coordinates. Local CM orientations were calculated from the intensity gradient of the cTnT image and mean orientation and alignment were quantified using spherical statistics[12]. All subsequent analysis was implemented in Matlab (Mathworks, Natick, MA).

For a given cTnT image I, the intensity gradient vector at each voxel, $\mathbf{G} = [G_x,G_y,G_z]^T$, was calculated by convolution with kernel h. We used a σ value of 5 μm for this study. The kernel size (s) was chosen such that the minimum value of h was 0.01, so that $-s \leq x,y,z \leq s$.

$$G_x = h_x * I, \ \ G_y = h_y * I, \ \ G_z = h_z * I \tag{1}$$

$$h_i(x,y,z) = \frac{2i}{\sigma^2}\exp\left(-\left(x^2+y^2+z^2\right)/\sigma^2\right), \ \ i \in \{x,y,z\} \tag{2}$$

To calculate local CM orientation, we divided the image into subregions of size *m* x *m* x *m*. All of the gradient vectors within a subregion ($\mathbf{G}^m$) were collected and treated as a girdle distribution. A girdle distribution of axes is concentrated around a great circle; it is an equatorial distribution[12]. The polar axis of a girdle distribution is the vector normal to the equatorial plane. As image gradient vectors are normal to the surfaces of objects, the polar axis is thus the dominant orientation of objects within the local window. The polar axis is the eigenvector associated with the smallest eigenvalue of the orientation matrix **T**[12].

$$\mathbf{T} = \begin{bmatrix} \sum \mathbf{G}_x^m \mathbf{G}_x^m & \sum \mathbf{G}_x^m \mathbf{G}_y^m & \sum \mathbf{G}_x^m \mathbf{G}_z^m \\ \sum \mathbf{G}_y^m \mathbf{G}_x^m & \sum \mathbf{G}_y^m \mathbf{G}_y^m & \sum \mathbf{G}_y^m \mathbf{G}_z^m \\ \sum \mathbf{G}_z^m \mathbf{G}_x^m & \sum \mathbf{G}_z^m \mathbf{G}_y^m & \sum \mathbf{G}_z^m \mathbf{G}_z^m \end{bmatrix} \qquad (3)$$

The selection of our window size *m* defines "local". Determining the window size requires some information about the size and shape of CM. We first defined a minimum window size, 30 μm in this study, based on the approximate average width of CM. The image was divided into subregions of this size and we rejected any subregion where the overall mean intensity was less than 5 times the global mean intensity of the entire image. For of each of the remaining initial subregions, we computed the orientation matrix eigenvalues ($\lambda_1 \le \lambda_2 \le \lambda_3$) and corresponding eigenvectors ($\mathbf{e}_1$, $\mathbf{e}_2$, $\mathbf{e}_3$) for window sizes between 30 μm and one-fourth the smallest image dimension, where the window center remained fixed and we increased the window size in 10 μm increments. We computed the shape parameter (γ) from the eigenvalues and selected the window size associated with the smallest γ value. Small γ values indicate blob or rod-shaped objects (likely CM) as opposed to plate-like objects[12]. The dominant local CM orientation for the window was then defined as the polar axis of the gradient vectors. For ME and ML ECTs, local CM orientations were converted from Cartesian to ECT coordinates based on the bundle centerline. The ECT coordinate was used for all further analysis.

$$\gamma = \frac{\ln\left(\lambda_3/\lambda_2\right)}{\ln\left(\lambda_2/\lambda_1\right)} \qquad (4)$$

Once we computed all of the local CM orientations, we treated the set as a Watson bipolar distribution. A Watson bipolar distribution is used for axial data, where the positive and negative directions of a vector are considered the same. We computed the principal axis as the eigenvector associated with the largest eigenvalue of the orientation matrix **T**. The alignment of CM was defined as the concentration of the distribution (κ), computed from eq. (5), where λ_3 is the largest eigenvalue of **T** and N is the total number of orientations[12]. A larger κ value indicates that the orientations are more concentrated along the principal axis. Concentrations were compared across ECT types using ANOVA to identify significant differences in CM alignment.

$$\lambda_3 / N = \int_0^1 x^2 \exp\left(\kappa x^2\right) dx \bigg/ \int_0^1 \exp\left(\kappa x^2\right) dx \qquad (5)$$

ECT Cellular Quantification

We quantified the CM or EC percentage in ECT bundles from 40X confocal images of DAPI and cTnT or CD31 stained tissue samples. We preprocessed DAPI images with a rolling ball background subtraction[13] and cTnT/CD31 images with contrast-limited adaptive histogram equalization[14]. DAPI and cTnT/CD31 images were then segmented using the Chan-Vese level set method[15]. After DAPI segmentation, nuclei were separated using a watershed and vertex graph algorithm[16]. For each nucleus, we determined CM or EC identity based on the overlap between the nucleus boundary and the segmented cTnT image.

RNA Extraction and Quantitative Reverse-Transcription Polymerase Chain Reaction (qPCR)

As previously described[17], fresh ECT samples were homogenised by an Omnitip Tissue homogenizer (USA Scientific, Ocala, USA; Cat. No. 6615-7273). Total RNAs were isolated using RNeasy Mini Kit (Qiagen, Valencia, USA; Cat. No. 74104) according to the manufacturer’s instructions. RNA quality and quantity were measured using the NanoDrop ND-2000 (Thermo Fisher Scientific). Reverse transcription was performed with the SuperScript VILO cDNA synthesis system (Invitrogen). TaqMan Gene Expression Assays (Best coverage and dose not detect gDNA) ordered from Thermo Fisher Scientific. qPCR was performed with StepOnePlus Real-time PCR system (Applied Biosystems). All qPCR were performed with biological triplicates for each group and 18S rRNA as an endogenous control.

Animal Model Preparation and ECT Implantation

All animal surgeries were performed following protocols approved by the University of Louisville Institutional Animal Care and the *Guide for the Care and Use of Laboratory Animals* prepared by the Institute for Laboratory Animal Research, U.S.A. (8th ed., 2011). Male athymic nude rats (NTac: NIH-Foxn1^{rnu}, Taconic Biosciences, Hudson, USA) weighing 270-340g were used as recipients for surgeries. One-half of ME-ECT was implanted in a normal nude rat to confirm the engraftment of the ECT on a rat heart through left thoracotomy. We then implanted an ME-ECT in a myocardial infarction (MI) model rat (Fig. 5a). MI was induced by permanent left anterior descending artery ligation using 7-0 silk suture as previously described[1,7]. Isoflurane (3-5%) inhalation was used for general anaesthesia, and subcutaneous or intraperitoneal injection of Buprenorphine (0.5mg/kg, twice a day, three days including operation day) was used for analgesia. ECT implantation was performed one week after MI induction during the “subacute phase” of MI. Left ventricular ejection fraction (EF) was evaluated six days after coronary artery ligation by echocardiography. Rats, whose hearts showed more than 60% EF or preserved more than 80% of initial EF, were excluded from subsequent experiments. A total of 10 rats were randomly divided into two groups: rats implanted with ECTs (Implant group; n=5) and sham-operated rats (Sham group; n=5). As previously described, the LV anterior wall was

exposed through redo-left thoracotomy[1,7]. Using 7-0 silk sutures, the anterior infarcted myocardium was covered with one whole ME-ECT, folded in half, along the LV circumferential direction. For the sham-operated group, a thoracotomy was performed one week after coronary ligation; however, no ECT implantation was performed (Fig. 4a). Hearts were harvested 4 weeks after treatment or sham operation and prepared for Masson's trichrome and immunohistochemistry.

Cardiac Functional Assessment

Transthoracic echocardiography was performed by an investigator blinded to group assignment using a high resolution Vevo2100 system (VisualSonics, Toronto, Canada) and 21-MHz imaging transducer (MS250; VisualSonics). Evaluations were performed before MI, ECT implantation (six days after MI induction), and two and four weeks after implantation. Stroke volume (SV) and ejection fraction was calculated by single plane area-length method. Cardiac index (CI) was calculated by the following formula: CI = SV x heart rate / body weight. Regional wall motion was traced, calculated and visualised using the VevoStrain application (VisualSonics)[18].

Histologic Measurement of Left Ventricular remodelling

To quantify the LV remodelling after MI with or without ECT implantation, five 8µm frozen sections at 400 µm to 500 µm intervals were stained with Masson's Trichrome. Sections were imaged under Olympus DP72 optical microscope (Olympus, Tokyo, Japan) and analysed using NIH ImageJ. Just like Tang's report, a series of morphometric parameters were measured in each section including total LV area, scar area, risk region area, LV wall thickness in the risk and non-infarcted regions, and LV expansion index. Risk area was defined as the LV area between the two edges of the infarct scar. Wall thickness was the average of 5 measurements equally distributed within risk and non-risk area. The LV expansion index was calculated from LV circumference and wall thickness to evaluate both LV dilation and wall thinning simultaneously: Expansion index = (endocardial circumference/epicardial circumference) x (non-infarcted region wall thickness/risk region wall thickness)[19].

Statistical Analysis

The data were analysed using JMP software for Windows (version10.0.2, SAS Institute Inc., Cary, NC). Results are presented as mean ± standard error of the mean (SEM). Comparisons between two groups were made with the unpaired t-test unless otherwise noted. Mann-Whitney U test was used for non-normal distributions. Comparisons between more than 2 groups were made with one-way or two-way repeated analysis of variance (ANOVA) followed by Tukey's test as post hoc. A p-value of less than 0.05 was considered significant.

References

1. Masumoto, H. *et al.* The myocardial regenerative potential of three-dimensional engineered cardiac tissues composed of multiple human iPS cell-derived cardiovascular cell lineages. *Sci. Rep.* **6,** 29933 (2016).

2. Takahashi, K. *et al.* Induction of pluripotent stem cells from adult human fibroblasts by defined factors. *Cell* **131,** 861–72 (2007).

3. Masumoto, H. *et al.* Human iPS cell-engineered cardiac tissue sheets with cardiomyocytes and vascular cells for cardiac regeneration. *Sci. Rep.* **4,** 6716 (2014).

4. Uosaki, H. *et al.* Efficient and Scalable Purification of Cardiomyocytes from Human Embryonic and Induced Pluripotent Stem Cells by VCAM1 Surface Expression. *PLoS One* **6,** e23657 (2011).

5. Bian, W., Liau, B., Badie, N. & Bursac, N. Mesoscopic hydrogel molding to control the 3D geometry of bioartificial muscle tissues. *Nat. Protoc.* **4,** 1522–34 (2009).

6. Tobita, K. *et al.* Engineered early embryonic cardiac tissue retains proliferative and contractile properties of developing embryonic myocardium. *Am. J. Physiol. Heart Circ. Physiol.* **291,** H1829-37 (2006).

7. Fujimoto, K. L. *et al.* Engineered fetal cardiac graft preserves its cardiomyocyte proliferation within postinfarcted myocardium and sustains cardiac function. *Tissue Eng. Part A* **17,** 585–96 (2011).

8. Matsuo, T. *et al.* Efficient long-term survival of cell grafts after myocardial infarction with thick viable cardiac tissue entirely from pluripotent stem cells. *Sci. Rep.* **5,** 16842 (2015).

9. Biot, E. *et al*. in *5th IEEE International Symposium on Biomedical Imaging: From Nano to Macro*. 975-978 (Paris, France, 2008).

10. Scientific Computing and Imaging Institute. 'Seg3D' Volumetric Image Segmentation and Visualization. *Scientific Computing and Imaging Institute (SCI*) <http://www.seg3d.org> (2015).

11. Schena, G. & Favretto, S. Pore space network characterization with sub-voxel definition. *Transp. Porous Med* **70,** 181–190 (2007).

12. Fisher, N. I., Lewis, T. & Embleton, B. J. J. Statistical analysis of spherical data. *Cambridge Univ. Press* (1987)

13. Sternberg, S. R. Biomedical Image-Processing. *Computer* **16,** 22–34 (1983).

14. Zuiderveld, K. Zuiderveld, K. *Graph. Gems IV* 474–485 (Academic Press, 1994)

15. Chan, T. F. & Vese, L. A. Active contours without edges. *IEEE T Image Process* **10,** 266–277 (2001).

16. Mouelhi, A., Sayadi, M. & Fnaiech, F. in *2011 International Conference on Communications, Computing and Control Applications (CCCA)* 1–6 (Hammamet, Tunisia, 2011)

17. Ye, F. *et al.* Gene expression profiles in engineered cardiac tissues respond to mechanical loading and inhibition of tyrosine kinases. *Physiol. Rep.* **1,** e00078 (2013).

18. Bauer, M. *et al.* Echocardiographic speckle-tracking based strain imaging for rapid cardiovascular phenotyping in mice. *Circ. Res.* **108,** 908–916 (2011).

19. Tang, X. L. *et al.* Intracoronary administration of cardiac progenitor cells alleviates left ventricular dysfunction in rats with a 30-day-old infarction. *Circulation* **121,** 293–305 (2010).

Supplementary Table

Group	n	CM (%)	EC (%)	MC (%)	UD (%)
**6M**	12	45.3±1.6	19.1±1.2	11.2±0.6	5.1±0.3
**9M**	7	44.3±1.2	17.3±1.6	11.5±0.8	5.1±0.4
**12M**	12	43.5±1.7	17.6±1.9	12.3±1.8	5.4±0.3
**12MH**	6	43.9±1.5	20.7±2.2	11.3±0.8	4.6±0.4

Supplementary Table 1: Initial cell compositions to investigate cell number and density. Percentage of cTnT, cardiac troponin-T $(cTnT)^+$ cardiomyocytes (CM), vascular endothelial (VE)-cadherin $(CD144)^+$ endothelial cells (EC), platelet-derived growth factor receptor beta (PDGFRβ; $CD140b)^+$ vascular mural cells (MC), TRA-1-60+ undifferentiated iPS cells (UD) prior to generating ECTs quantified by flow cytometry. Data are expressed as the mean ± SEM.

Supplementary Figures

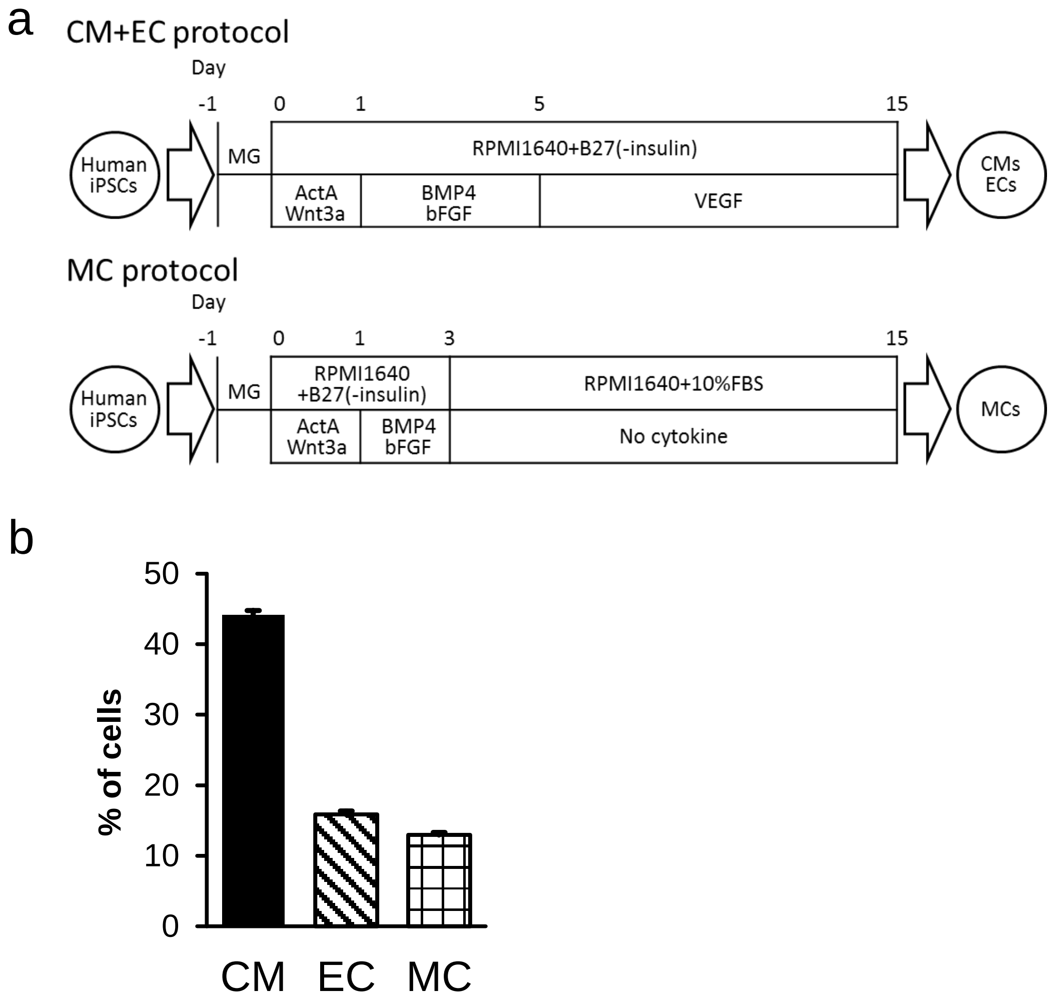

Supplementary Figure 1: Protocols to differentiate cardiovascular cell populations from human iPS cells. (a) Schematic diagrams of protocols used to induce cardiac lineage cells (CM+EC protocol, n=53, and MC protocol, n=53). CM, cardiomyocyte; EC, endothelial cell; MC, vascular mural cell; iPSC, induced pluripotent stem cell; MG, Matrigel; ActA, Activin A; Wnt3a, BMP4, Bone morphogenetic protein 4; bFGF, basic fibroblast growth factor; VEGF, vascular endothelial cell growth factor; FBS, fetal bovine serum. **(b)** Population of $cTnT^{+}$ CMs, VE-cadherin $(CD144)^{+}$ ECs and PDGFRβ $(CD140b)^{+}$ MCs for ECT preparation quantified by flow cytometry (n=107). cTnT, cardiac troponin-T; VE-caeherin, vascular endothelial-cadherin; PDGFRβ, platelet-derived growth factor receptor beta.

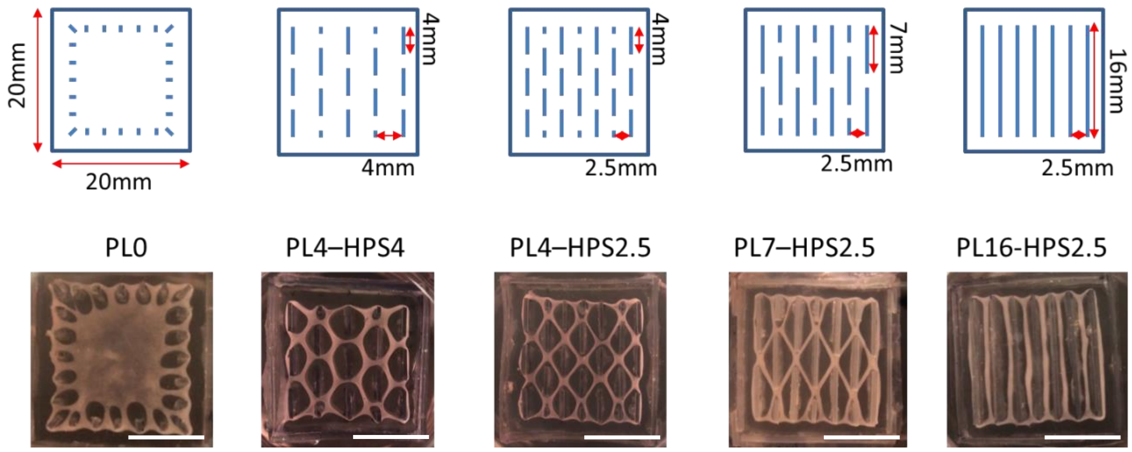

Supplementary Figure 2: Initial LF-ECT geometries considered to investigate the effect of construct geometry on tissue maturation and function. Upper row includes a range of mold templates: PL-ECT (peripheral posts only) and a range molds that vary the post length (PL 4 ,7, 16 mm) and post spacing (PS 4 or 2.5 mm). Lower row shows various representative LF-ECTs at d14 in their respective molds. Scale bar: 10 mm.

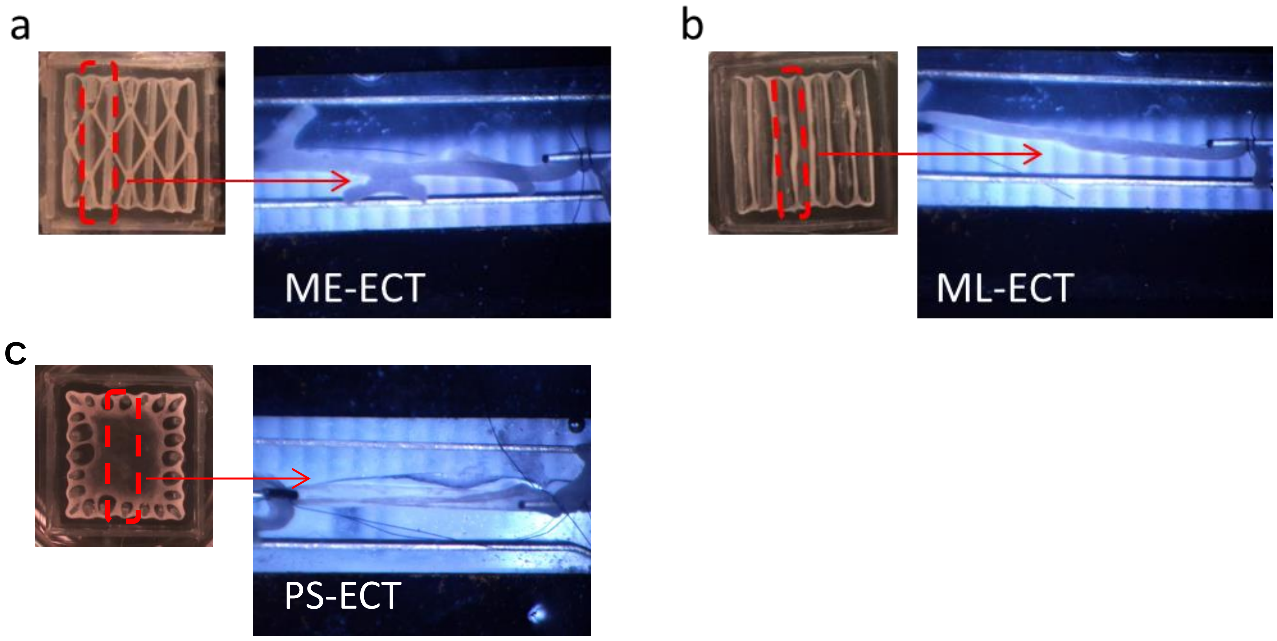

Supplementary Figure 3: Contractile force measurement. **(a)** Representative ME-ECT segment, which was cut off at the red dotted line, used to determine in vitro force-length and force-frequency relations. Note that the isolated ME-ECT construct includes junctions and remnants of adjacent bundles. ECTs are attached to the muscle testing system using 10-0 nylon suture. **(b)** Similar views are provided for an ML-ECT and **(c)** a PS-ECT construct.

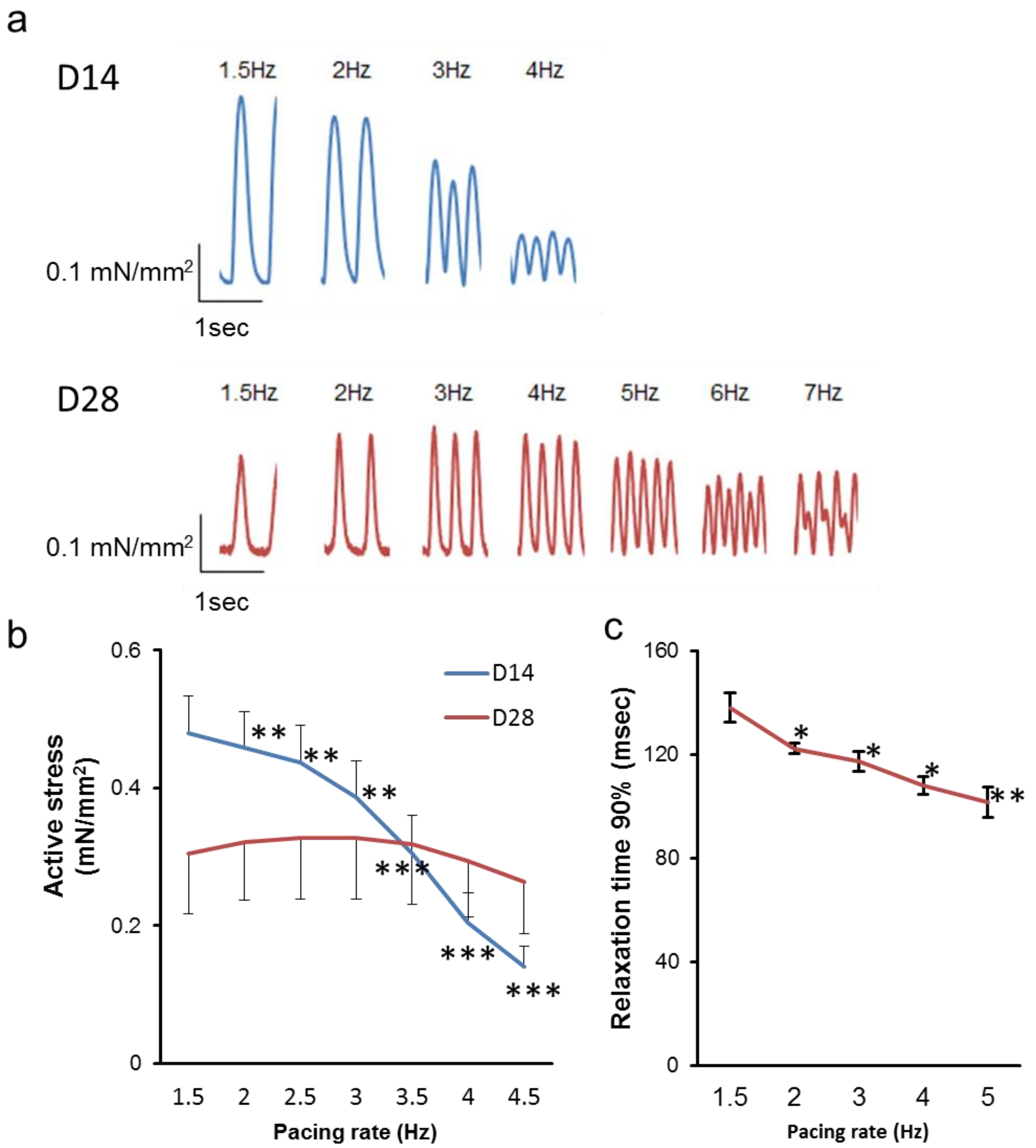

Supplementary Figure 4: Force-frequency relationship of ME-ECTs. (a) Representative waveforms of active stress at different pacing frequency in 14-day and 28-day constructs. **(b)** The actual value of active stress at each beating rate (n=6; **p<0.01; ***p<0.001 vs baseline at 1.5Hz in D14). **(c)** 28-day constructs showed frequency-dependent acceleration of relaxation (n=6; *p<0.05; **p<0.01** vs baseline at 1.5Hz in D28).

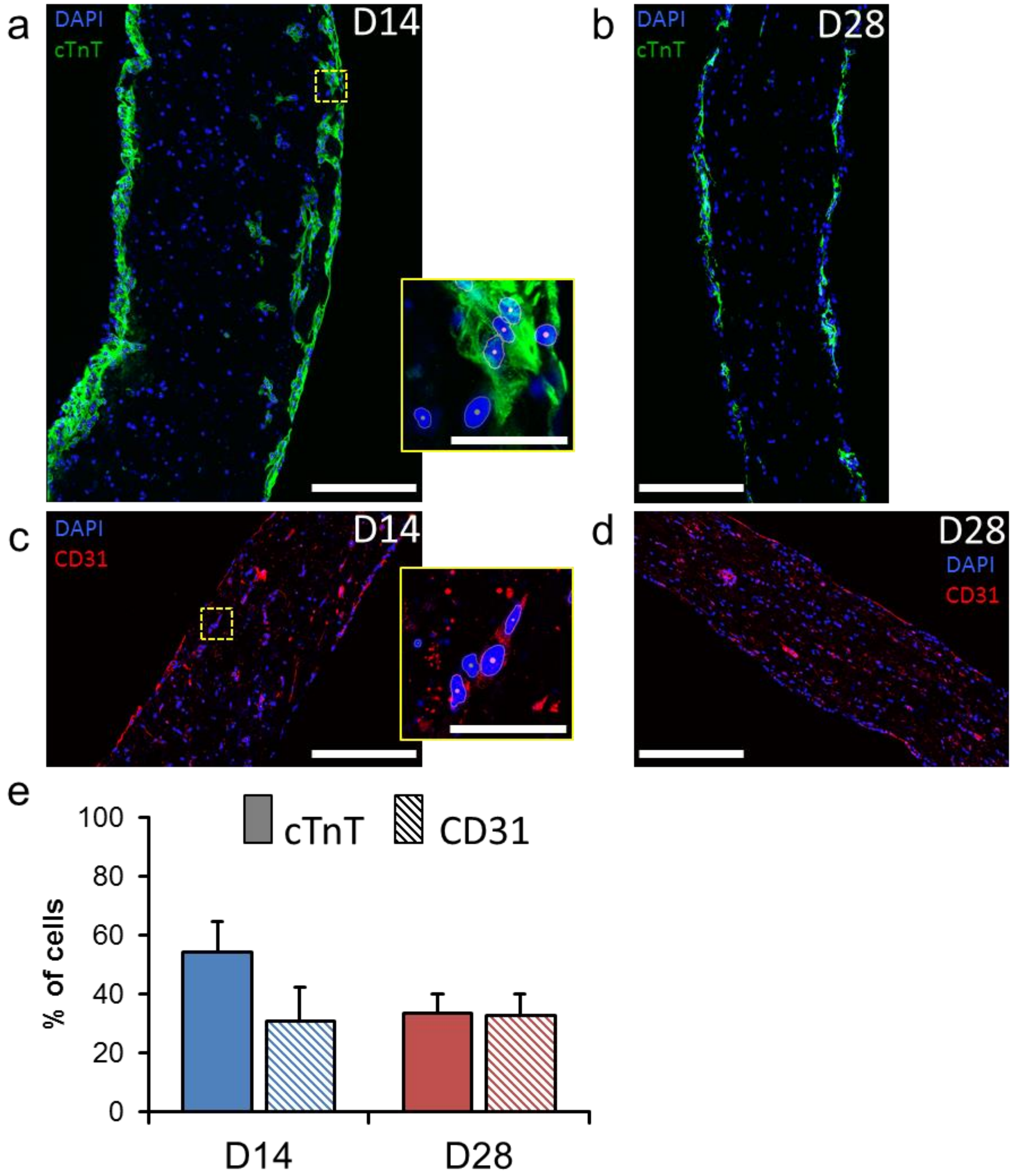

Supplementary Figure 5: Cardiomyocyte and endothelial cell populations in LF-ECT bundles. (a,b) Representative cross-sectional images of DAPI (blue) and cTnT (cardiac Troponin T, green) in **(a)** 14-day and **(b)** 28-day LF-ECT bundles. The inset in **(a)** shows the nuclei classification for the region marked by the dashed yellow box. cTnT positive nuclei are outlined in orange and negative nuclei are outlined in grey. **(c,d)** Representative cross-sectional images of DAPI (blue) and CD31 (red) in 14-day (c) and 28-day (d) LF-ECT bundles. The inset in **(c)** shows the nuclei classification for the region marked by the dashed yellow box. CD31 positive nuclei are outlined in pink and negative nuclei are outlined in grey. Scale bars: 250 µm, 50 µm inset. **(e)** The percent positive cTnT and CD31 cell fractions in day 14 and day 28 LF-ECTs (n=4).

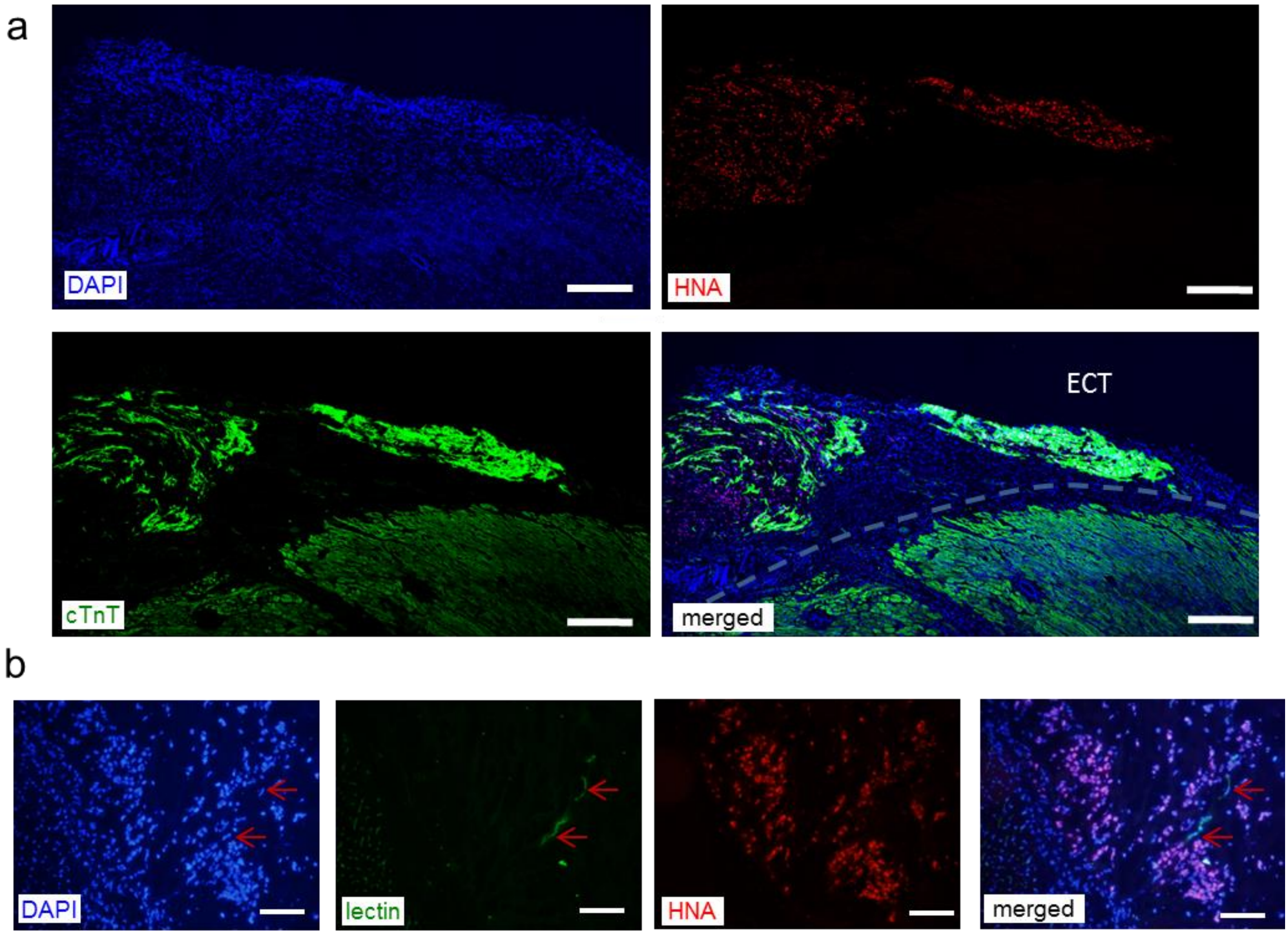

Supplementary Figure 6: ME-ECT survival and vascular coupling. **(a)** Representative left ventricular histology 4 weeks after implantation of hiPSC-ME-ECT onto non-infarcted heart. Grey dotted line indicates the transition from implanted ME-ECT to recipient myocardium. DAPI, nuclear stain; HNA, human nuclear antigen; cTnT, cardiac Troponin T; merged image. Scale bar: 250 µm**.** **(b)** Higher magnification within ME-ECT showing evidence for perfusion by injected lectin (red arrows). DAPI, nuclear stain; lectin, intravascular perfusion; HNA, human nuclear antigen; merged image. Scale bar: 125 µm**.**

Supplementary Video

Supplementary Video 1: Representative cardiomyocyte (CM) distribution in a day 14 ME-ECT bundle and junction. Whole mount ME-ECT stained to identify CM (cardiac Troponin T, green; DAPI, nuclear stain, blue). Note that CM density is greatest on the ME-ECT surface.
